# Nomenclature and Comparative Morphology of the Teneurin/TCAP/ADGRL Protein Families

**DOI:** 10.3389/fnins.2019.00425

**Published:** 2019-05-03

**Authors:** Luciane V. Sita, Giovanne B. Diniz, José A. C. Horta-Junior, Claudio A. Casatti, Jackson C. Bittencourt

**Affiliations:** ^1^Laboratory of Chemical Neuroanatomy, Department of Anatomy, Institute of Biomedical Sciences, University of São Paulo, São Paulo, Brazil; ^2^Department of Anatomy, Institute of Biosciences, São Paulo State University, São Paulo, Brazil; ^3^Department of Basic Sciences, São Paulo State University, São Paulo, Brazil; ^4^Center for Neuroscience and Behavior, Department of Experimental Psychology, Institute of Psychology, University of São Paulo, São Paulo, Brazil

**Keywords:** TEN, Odz, ADGRL, latrophilin, teneurin C-terminal associated peptide

## Abstract

The teneurins are a family of glycosylated type II transmembrane proteins synthesized in several tissue from both vertebrate and invertebrate species. These proteins interact with the latrophilins, a group of adhesion G protein-coupled receptors. Both teneurins and latrophilins may have been acquired by choanoflagellates through horizontal gene transfer from a toxin-target system present in prokaryotes. Teneurins are highly conserved in eukaryotes, with four paralogs (TEN1, TEN2, TEN3, and TEN4) in most vertebrates playing a role in the normal neural development, axonal guiding, synapse formation and synaptic maintenance. In this review, we summarize the main findings concerning the distribution and morphology of the teneurins and latrophilins, both during development and in adult animals. We also briefly discuss the current knowledge in the distribution of the teneurin C-terminal associated protein (TCAP), a peptidergic sequence at the terminal portion of teneurins that may be independently processed and secreted. Through the analysis of anatomical data, we draw parallels to the evolution of those proteins and the increasing complexity of this system, which mirrors the increase in metazoan sensory complexity. This review underscores the need for further studies investigating the distribution of teneurins and latrophilins and the use of different animal models.

## Introduction and Nomenclature

The teneurins are a family of glycosylated type II transmembrane proteins synthesized in several tissue from both vertebrate and invertebrate species (Tucker et al., 2012). The nervous system has been conserved as the main site of teneurins synthesis in a variety of species, where teneurins are prevalent in neuronal projections (Oohashi et al., 1999; Lovejoy et al., 2006). As a type II transmembrane protein, the teneurins have a simple amino terminus located on the cytoplasmic side of the cell, while a carboxy terminus rich in binding motifs is located outside the cell, including an epidermal growth factor (EGF)-like domain, which contains a region of conserved cysteine residues and a stretch of tyrosine-aspartate-repeats (Rubin et al., 1999; Tucker and Chiquet-Ehrismann, 2006; Young and Leamey, 2009). The carboxy-terminal sequence of teneurins, spanning approximately 40 residues and located in the last exon, is flanked by a dibasic cleaving motif and an amidation motif on the carboxy terminal, suggesting this sequence can be cleaved and amidated, generating a small biologically active peptide. This processed peptide has been called teneurin C-terminal associated peptide (TCAP) (Qian et al., 2004; Lovejoy et al., 2006). Mounting evidence suggests that some TCAP paralogs can be processed independently of the main peptide, reinforcing the idea that these small molecules may act as neuronal messengers (Chand et al., 2013). Both teneurins and TCAPs are believed to interact with latrophilins, a family of G-protein coupled receptors (GPCRs) of the adhesion family (Lelianova et al., 1997; Husić et al., 2019). This transsynaptic complex appears to participate in axonal guiding, synapse formation and synaptic maintenance. To do that, teneurins, TCAPs and latrophilins also interact with other membrane proteins, such as fibronectin leucine-rich transmembrane (FLRT), neurexins and dystroglycans (as reviewed in Woelfle et al., 2015). In this review, we will evaluate the available morphological information about the teneurins and latrophilins, highlighting the scarcity of available information about these proteins.

### Family History

**FIGURE 1:**
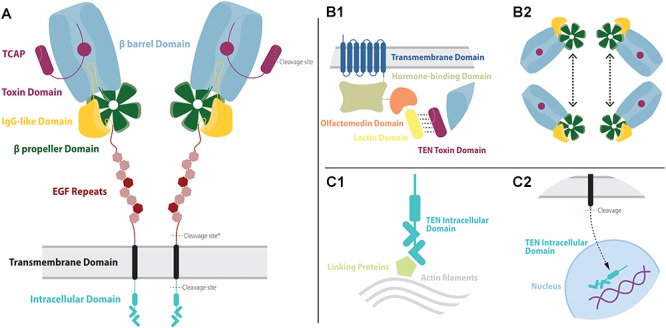
The overall structure of teneurins and their molecular partners. **(A)** Teneurins are composed of seven major domains: intracellular domain (teal), transmembrane domain (black), 8 EGF repeats (red), an IgG-like domain (yellow), a β propeller domain (green), a β barrel domain (blue) and a toxin domain (purple). Among the EGF repeats are two modified copies, repeats 2 and 5 (darker red). The EGF stem is important for the dimerization of teneurins in the presynaptic membrane. The IgG-like and β propeller domains help seal the β barrel domain from underneath. The β barrel domain partially surrounds the N-terminal portion of the toxin domain, but a gap in its wall allows some of the toxin domain to extend outwards. At the C-terminal portion of the toxin domain is the processable sequence called the teneurin C-terminal associated protein, or TCAP. Three major cleavage sites are found in the teneurin structure. One cleavage site is located in the intracellular domain and allows this portion to be processed and translocated to the nucleus. Exclusively in teneurin 2, a cleavage site (marked with an asterisk) allows the whole extracellular portion of the protein to be released in the extracellular space. Finally, a cleavage site inside the toxin domain allows the release of TCAP. **(B1)** Extracellularly, the teneurins are able to interact with ADGRLs, a class of adhesion GPCRs. The binding of teneurin and latrophilins depends mainly on the interaction between the outer portion of the toxin domain and the lectin domain of ADGRLs. **(B2)** Teneurins are also able to interact with other teneurins. Essential for that interaction is the β propeller domain, who strongly binds similar pairs of teneurins. **(C1)** Intracellularly, teneurins can bind to actin filaments of the cytoskeleton through linker proteins. **(C2)** In certain conditions, the intracellular domain of teneurins can be cleaved and translocated to the nucleus to act as a transcription modulator.

Recent works suggest teneurin homologs first originated from a prokaryotic transmembrane polymorphic proteinaceous toxin gene, which was incorporated to the genome of the choanoflagellate *Monosiga brevicollis* (Tucker et al., 2012; Tucker, 2013). This theory is supported by the presence of proteins with a similar structure to the extracellular domain of the *Monosiga* teneurin in aquatic bacteria while other non-metazoan opisthokonts lack similar proteins, and by the frequent occurrence of horizontal gene transfer in this species (Tucker, 2013). It is believed that this prokaryotic toxin gene then merged with an EGF-like domain repeats-rich gene, forming a protein similar in structure to the modern teneurin homologs. Among the structural similarities between the *Monosiga* and the metazoan teneurins are eight EGF repeats, a cysteine rich domain, and a partially similar (YD)-repeat motif (Tucker, 2013). The general structure of teneurins is illustrated in Figure 1. Since choanoflagellates are the closest relatives of metazoans (King et al., 2008), it is likely that this event of horizontal gene transfer was then conserved upon the emergence of the metazoan due to the teneurins actions to promote normal development (Tucker et al., 2007; Trzebiatowska et al., 2008).

**FIGURE 2:**
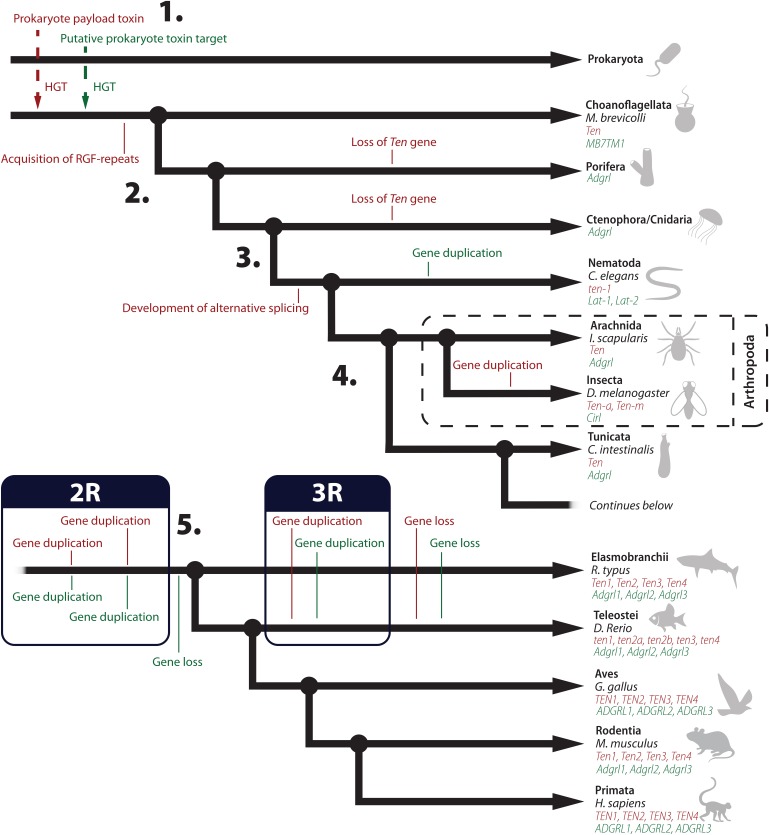
The main events in the phylogenetic history of teneurins and ADGRLs. Some clades were omitted for clarity. Events pertaining to teneurins are indicated in red and those related to the ADGRLs are indicated in green. (1) The likely origin of the teneurin-latrophilin system is a Horizontal Gene Transfer (HGT) from prokaryotes to choanoflagellates, as evidenced by the presence of teneurin-like and a latrophilin-like proteins in *Monosiga brevicolli*. (2) With the emergence of metazoa, the teneurin gene was lost in Porifera and Ctenophora/Cnidaria, since no homologous gene can be found on these species. It is likely that such loss occurred independently on those clades, instead of occurring at the root of the metazoan tree. It should be noticed that both clades retained *Adgrl*-like genes, possibly due to functions played by these receptors that are independent of teneurins, what led to their conservation. (3) A series of events occurred at the emergence of the Nematoda clade. First, these species are the first known to possess alternative promoters that drive different isoforms of teneurin, with distinct patterns of distribution. It is likely that this facilitated the conservation and acquisition of new functions by teneurins paralogs that originated later. In the nematode lineage a gene duplication of the *Adgrl* gene also occurred, resulting in two different latrophilin-like proteins (*Lat-1* and *Lat-2*). (4) A duplication of the teneurin gene occurred after the divergence of the Insecta clade, with several dipterans reported to contain two copies of *Ten*, identified as *Ten-a* and *Ten-m* in the most studied animal model of this clade, *D. melanogaster*. (5) The final major phylogenetic event concerning the teneurin-latrophilin system occurred at the emergence of the vertebrata clade. While tunicates have a single copy of *Ten* and *Adgrl*, vertebrates have four paralogs of *Ten* (*Ten-1* through *Ten-4*) and three paralogs of *Adgrl* (*Adgrl1* through *Adgrl3*). The double duplication of teneurins at the root of vertebrates fits well with the 2R hypothesis of a double whole genome duplication occurring at this timepoint. A subsequent loss of one of the newly formed *Adgrl* paralogs must have occurred to result in the current observable three paralogs of this gene in vertebrates. Exclusively in the Actinopterygii lineage, an additional whole genome duplication occurred, followed by successive gene losses, resulting in 5 paralogs of teneurins in modern day teleosts.

Complete or partial teneurin sequences have been found in trematodes (*Schistosoma mansoni*), nematodes (*Caenorhabditis elegans*), annelids (*Capitella teleta*), mollusks (*Lottia gigantea*), arthropods (*Apis mellifera*, *Tribolium castaneum*, *Aedes aegypti*, *Culex quinquefasciatus*, *Drosophila melanogaster*, *Daphnia pulex*, and *Ixodes scapularis*) and the purple sea urchin (*Strongylocentrotus purpuratus*). There is no homologous gene found in the sequences from sponges, placozoans, ctenophores, cnidarians, fungi, ichthyospores or nucleariids (Tucker et al., 2012). As a whole, these results suggest the acquisition of the teneurin gene in the choanoflagellate, its loss in some clades (Porifera, Ctenophora, Placozoa, and Cnidaria) and its retention in bilaterians, what is illustrated in Figure 2.

Most non-chordate bilaterians have a single teneurin gene (*Ten*), including the well-studied animal model *C. elegans*. Two genes are found in *D. melanogaster*, as well as in several other insect species (honey bee, flour beetle, mosquitoes). The same, however, is not observed for other arthropods, such as the crustacean *D. pulex* and the arachnid *I. scapularis*, suggesting that a gene duplication occurred after the divergence of the *ectognatha* clade (Tucker et al., 2012). These observations, coupled to the fact that the protochordates *Ciona intestinalis* and *Branchiostoma floridae* have a single Ten-coding gene in their genome, while the elasmobranch *Rhincodon typus* (whale shark) has four predicted *Ten* paralogs (*Ten1*, *Ten2*, *Ten3*, and *Ten4*), suggests that the two rounds (2R) of whole genome duplication that likely occurred in the early vertebrate lineage (Dehal and Boore, 2005; Ohno, 2013; Sacerdot et al., 2018) account for the diversification of teneurin paralogs.

The teleost fish *Danio rerio* has five different *Ten* genes, identified as *Ten1*, *Ten2A*, *Ten2B*, *Ten3*, and *Ten4* (Tucker et al., 2012). Another teleost, the stickleback *Gasterosteus aculeatus*, also has five *Ten* paralogs, but with a distinct set: *Ten1*, *Ten2*, *Ten3A*, *Ten3B*, and *Ten4* (Tucker et al., 2012). This observation fits well with the 3R theory (Meyer and Van de Peer, 2005), with another whole genome duplication round taking place in the *actinopterygii* lineage, resulting in eight *Ten* genes, followed by the fast loss of some of these genes in individual teleost lineages. Finally, chicken (*Gallus gallus*), mice (*Mus musculus*), brown rats (*Rattus norvegicus*), and humans (*Homo sapiens*) have each four *Ten* paralogs, suggesting those early duplications were largely maintained during vertebrate evolution (Tucker et al., 2012).

### The Name of the Game

As it has occurred with most bioactive substances, the discovery and the description of teneurins did not follow any kind of phylogenetic reasoning. This led to a somewhat convoluted nomenclature. The first description of teneurins was made in *D. melanogaster* by two different groups. Baumgartner and Chiquet-Ehrismann (1993), searching for invertebrate homologs of the vertebrate extracellular matrix glycoprotein tenascin, identified a gene that coded for a protein with a partially similar structure, which they called the tenascin-like molecule accessory, or *ten^a^*/Ten^a^. Looking for other tenascin-like sequences in the *Drosophila* genome, Baumgartner et al. (1994) found a second sequence, which they called the tenascin-like molecule major, *ten^m^*/Ten^m^. This second protein was located to odd segments during *Drosophila* development, with mutants exhibiting a pair-ruled phenotype. In the same year, Levine et al. (1994) discovered a gene (and its protein) that was also expressed in odd-segments of the *Drosophila* embryo, was not a transcription factor, and had EGF-like repeats that linked this protein to the vertebrate tenascin. They called this gene odd Oz (*odz*), and its protein Odz. We now know that Ten^m^ and Odz are the same protein, and despite the significant similarity between Ten^m^ EGF-like repeats and that of the tenascins, they are structurally and functionally distinct, forming their own family of proteins.

The first vertebrate homolog of the tenascin-like proteins was discovered 4 years later, by Wang et al. (1998). These researchers were searching for genes that were differentially expressed upon stress response in the endoplasmic reticulum of mice. One of the identified genes, called DOC4 (after downstream of CHOP 4), coded for a protein that showed 31% identity and 50% similarity to *Drosophila* Ten^m^/Odz, and its expression pattern in the developing mouse embryos resembled that described for the invertebrate counterpart. These authors also described the existence of a homologous gene to DOC4 in the human genome, the first human teneurin ortholog described.

The discovery of DOC4 was followed by the description of several vertebrate orthologs. Oohashi et al. (1999) made an important advance in our understanding of teneurins describing four mouse genes that are similar to the Drosophila *ten^m^*. These genes and their protein were then termed ten-m1/Ten-m1, ten-m2/Ten-m2, ten-m3/Ten-m3 and ten-m4/Ten-m4. These authors also showed that ten-m4 was identical to the previously described DOC4. Concomitantly, Ben-Zur and Wides (1999) and Ben-Zur et al. (2000) also described four mouse orthologs of *ten^m^*, using the *Odz1* through *Odz4* nomenclature. In that same year, Minet et al. (1999) and Rubin et al. (1999) described the first avian *ten^m^* homologs, which they called teneurin-1 and teneurin-2, referencing both the phylogenetic history of this protein and the main site of expression of this gene in the chicken. Mieda et al. (1999) identified two *ten^m^* homologs in zebrafish, which they called *ten-m3* and *ten-m4* due to their correspondence to the recently described mouse genes.

**FIGURE 3:**
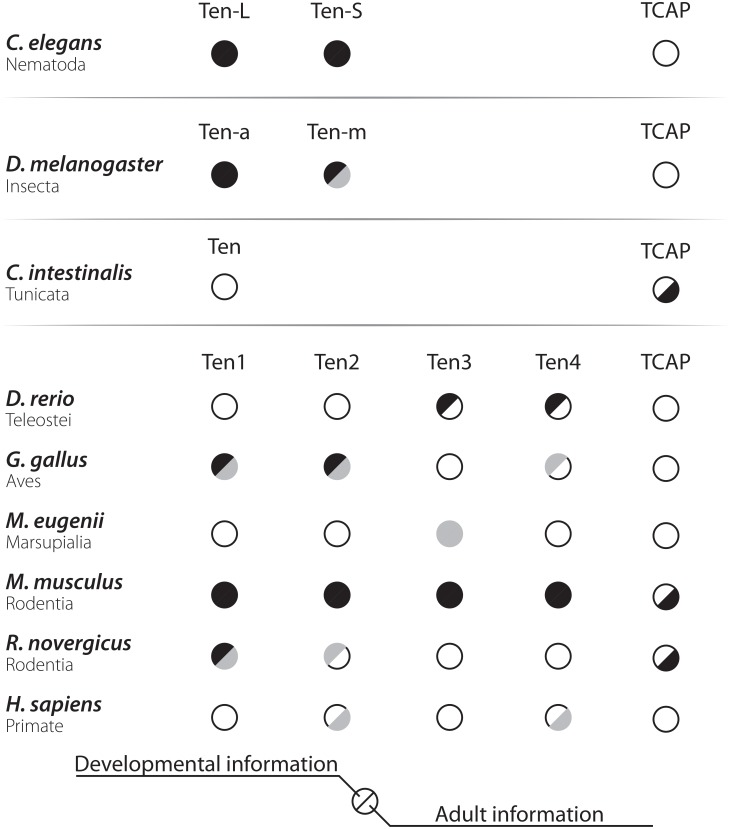
Visual representation of the available information about the morphology of teneurin system in different species. Empty half-circles represent complete lack of information, gray half-circles represent partial or incomplete information, and full half-circles represent complete and abundant description. The left half-circle represents developmental information, and the right side represents adult information. Several gaps in our knowledge about the morphology of the teneurin system can be seen in the graph. Besides the invertebrate metazoans, only the lab rodent *Mus musculus* has what can be described as a complete anatomical description. Another popular lab rodent, *Rattus norvegicus*, has partial information available during its development and next to nothing in the adult. Since mice and rats diverged over 10 million years ago, comparisons between these two species could be informative in terms of plasticity of the teneurin system. Detailed mappings performed in rats are still necessary to allow these comparisons, however. The marsupial *Macropus eugenii* has been used solely for descriptions of *Ten3* in the visual system and could represent an interesting animal model for the study of the teneurins. In a similar vein, the information available for teleost species is scarce and should be obtained with high priority. This information could help us understand the emergent complexity of paralogs originated at the vertebrate lineage and the specific duplications of the *Ten* gene that occurred in this clade could inform us about the retention of newly generated proteins. The amount of information for primates can also be improved, as only punctual data is available for teeth and ovaries from human origin. This lack of works in humans can be attributed to the difficulties in obtaining and working with these tissues, but such barriers can be overcome through the use of other primate models, such as the *Rhesus* or the *Sapajus* monkeys. Finally, more information about TCAP and its distribution must be obtained for virtually every species known.

While investigating the second extracellular loops of odorant receptors, Otaki and Firestein (1999) identified a rat homolog of *ten^m^*, which they called neurestin. Meanwhile, Minet and Chiquet-Ehrismann (2000) described the four human teneurins. In this work, we will employ a nomenclature that mostly follows that proposed by Minet and Chiquet-Ehrismann (2000), with a minor change: *Drosophila* genes will be noted as *Ten-a* and *Ten-m*, following the rules for *Drosophila* genes and the entry of these genes on the available databanks. Figure 3 summarizes the available knowledge about teneurin orthologs/paralogs in terms of their distribution during development and maturity in different organisms. Supplementary Tables S1 and S2 compile the different antibodies and probes used in the literature to investigate the distribution of members of the Teneurin family of proteins. Table 1 summarizes the nomenclature employed in this work when referring to the teneurin system.

**Table 1:** Nomenclature of the teneurin system.

Protein name	Protein abbreviation	Gene abbreviation	Synonymous notations	First use
***M. brevicolli***				
Teneurin	TEN	*Ten*		
***C. elegans***				
Teneurin	TEN-S/TEN-L	*ten-1*	R13F6.4	Wilson et al., 1994
***D. melanogaster***				
Tenascin-like molecule major	TEN-m	*Ten-m*	*ten^m^*/Ten^m^	Baumgartner et al., 1994
			*odz/*Odz	Levine et al., 1994
Tenascin-like molecule accessory	TEN-a	*Ten-a*	*ten^a^*/Ten^a^	Baumgartner and Chiquet-Ehrismann, 1993
***C. intestinalis***				
Teneurin-1	Ten1	*Ten1*		Colacci et al., 2015
***D. rerio***				
Teneurin-1	Ten1	*ten1*		
Teneurin-2a	Ten2a	*ten2a*		
Teneurin-2b	Ten2b	*ten2b*		
Teneurin-3	Ten3	*ten3*	ten-m3	Mieda et al., 1999
Teneurin-4	Ten4	*ten4*	ten-m4	Mieda et al., 1999
***G. gallus***				
Teneurin-1	TEN1	*TEN1*	ten-1	Minet et al., 1999
Teneurin-2	TEN2	*TEN2*	ten-2	Rubin et al., 1999
Teneurin-3	TEN3	*TEN3*	ten-3	
Teneurin-4	TEN4	*TEN4*		Tucker et al., 2000
***M. eugenii***				
Teneurin-1	TEN1	*TEN1*		
Teneurin-2	TEN2	*TEN2*		
Teneurin-3	TEN3	*TEN3*		
Teneurin-4	TEN4	*TEN4*		
***M. musculus***				
Teneurin-1	TEN1	*Ten1*	ten-m1	Oohashi et al., 1999
			odz-3	Ben-Zur and Wides, 1999
			odz-1	Ben-Zur et al., 2000
Teneurin-2	TEN2	*Ten2*	ten-m2	Oohashi et al., 1999
			odz-1	Ben-Zur and Wides, 1999
			odz-2	Ben-Zur et al., 2000
Teneurin-3	TEN3	*Ten3*	ten-m3	Oohashi et al., 1999
			odz-2	Ben-Zur and Wides, 1999
			odz-3	Ben-Zur et al., 2000
Teneurin-4	TEN4	*Ten4*	DOC4	Wang et al., 1998
			ten-m4	Oohashi et al., 1999
			odz-4	Ben-Zur and Wides, 1999
***R. norvegicus***				
Teneurin-1	TEN1	*Ten1*		
Teneurin-2	TEN2	*Ten2*	Neurestin	Otaki and Firestein, 1999
Teneurin-3	TEN3	*Ten3*		
Teneurin-4	TEN4	*Ten4*		
***H. sapiens***				
Teneurin-1	TEN1	*TEN1*		Minet and Chiquet-Ehrismann, 2000
Teneurin-2	TEN2	*TEN2*		Minet and Chiquet-Ehrismann, 2000
Teneurin-3	TEN3	*TEN3*		Minet and Chiquet-Ehrismann, 2000
Teneurin-4	TEN4	*TEN4*		Minet and Chiquet-Ehrismann, 2000


*The table presents a collection of synonymous presented in the literature for the teneurin orthologs/paralogs found in different species and the corresponding protein and gene abbreviations*.

### The Sorcerer’s Hat

A striking feature of the teneurins is the presence of hallmarks associated to bioactive substances in the 40 or 41 C-terminal residues. This sequence was discovered when Qian et al. (2004) screened a hypothalamic cDNA library of the rainbow trout (*Oncorhynchus mykiss*) for paralogous genes of the CRF family. Upon their search, one of the clones identified was the trout ortholog of *ten3*. The predicted C-terminal region encoded in the last exon of *ten3* had a neuropeptide-like structure with low sequence similarity to the CRF family of peptides, hence why this gene was cloned during their search. Furthermore, a predicted cleavage motif in the N-terminal of that putative peptide and an amidated carboxy terminal also contribute to the idea that this peptide could be synthesized, processed and release as a bioactive substance. The authors named it the teneurin C-terminal associated peptide (TCAP)-3. Despite the similarity between TCAP and CRF, there is no clear evidence that these two peptides share a common phylogenetic history. It is likely that they were introduced at different times in the metazoan lineage, and the three-dimensional structure of TCAP is highly dissimilar to that of CRF (Chen et al., 2013; Li et al., 2018).

Since a cleavage signal can be found in all paralogs of vertebrate teneurins, the identification of TCAP-3 by Qian et al. (2004) allowed the inference that TCAP-1, TCAP-2, and TCAP-4 could all be potentially synthesized and released as bioactive substances in vertebrates. TCAP orthologs were soon identified in mice and humans by Wang et al. (2005). Since both *Ten-m* and *Ten-a* have related peptide sequences to TCAP in their C-terminal portions (Lovejoy et al., 2006), it is likely that TCAP is a ubiquitous peptide for animals that have teneurin genes and may represent the modern equivalent of the ancient toxin payload of prokaryotes. Notably, all studies so far have used a synthetic formulation of TCAP based on the predicted sequence and structure of mouse TCAP1 (Wang et al., 2005). The isolation and purification of TCAP, therefore, could represent an interesting source of information about TCAP and its mechanisms of action.

A major advance in the understanding of the TCAP system came with the discovery that TCAP-1 may be synthesized independent of *Ten1*, with several lines of evidence supporting this idea. First, a short transcript of 600 base pairs corresponding to the last exon of *Ten1* (exon 31) can be identified in whole mouse brain extracts by northern blot, in addition to the whole *Ten1* transcript of approximately 8,000 base pairs. This short transcript could either be the result of independent gene expression or a highly specialized alternative splicing, where only the last exon of the protein is transcribed. Probes directed to other exons result only in the detection of the full-length transcript. Western blot analysis corroborate the presence of short proteins with molecular weights compatible with the independent expression of a segment of exon 31 corresponding to TCAP. In addition, TCAP-1 and Ten1 occupy largely different subcellular compartments, with both TCAP-1 and Ten1 found in the surface of cells in culture, while only TCAP-1 is found in the cytosol. Finally, the distribution of *Ten1* mRNA and the mRNA corresponding to the TCAP-1 portion are expressed in a distinct pattern in some regions of the brain, while overlapping in others (Zhou et al., 2003; Wang et al., 2005; Chand et al., 2013). This potentially decouples TCAP-1 and *Ten1* in the rodent CNS. A similar mechanism may take place with TCAP-3, but evidence is lacking (Woelfle et al., 2015). On the other hand, TCAP-2 and TCAP-4 are believed to have their synthesis coupled to their respective teneurins (Woelfle et al., 2015). Therefore, the distribution of *Ten2*, *Ten3*, and *Ten4* will be used as proxy to determine the distribution of TCAP-2, TCAP-3, and TCAP-4, while the distribution of *Ten1* and TCAP-1 will be examined separately, when data is available.

### Toxic Origins

The adhesion G protein-coupled receptor L (ADGRL) family, also known as latrophilins, is a family of G Protein-Coupled Receptors (GPCR) belonging to the adhesion family of GPCRs (Silva et al., 2011). These receptors were initially identified as ligands for the black widow spider toxin component, α-latrotoxin (Lelianova et al., 1997). This is evocative of the teneurin origin, as they are thought to be originated from a prokaryotic toxin gene, making the ancient ADGRL orthologs the possible targets for that toxin. This idea is further strengthened by the indication that ADGRLs may also have evolved by lateral gene transmission from prokaryotes to metazoan ancestors (Zhang et al., 2012).

There are three paralogs of ADGRLs, termed ADGRL1, ADGRL2, and ADGRL3. As is the case with the teneurins, the ADGRLs also received multiple names depending on the group, species and publication. In the original discovery of ADGRLs, by Davletov et al. (1996) and Lelianova et al. (1997), the rat receptor to α-latrotoxin was termed latrophilin 1, and its abbreviation LPH1. In parallel, an independent group also identified α-latrotoxin ligands that were independent of calcium, which they called CIRL, for Calcium-Independent Receptor of α-Latrotoxin (Krasnoperov et al., 1996, 1997). Following, the protein was isolated from mouse synaptosomes (Ichtchenko et al., 1998) and called CIRL/latrophilin (CL1). Soon, the same group described the protein paralogs, which received the names CIRL/latrophilin (CL1-3) (Sugita et al., 1998), latrophilin-2 (LPH-2) and latrophilin (LPH3) and CIRL-2 and CIRL-3 (Ichtchenko et al., 1999; Matsushita et al., 1999).

In 2004, orthologs of the ADGRLs were discovered in *C. elegans*, where they were called lat-1/LAT-1 and lat-2/LAT-2 (Mee et al., 2004; Willson et al., 2004; Langenhan et al., 2009). Some authors employ the abbreviation Lphn1–Lphn3 for the protein, and Adgrl1-Adgrl3 for the corresponding gene (Lu et al., 2015; Anderson et al., 2017). Others also use the abbreviation ADGRL for the proteins (Leon et al., 2017). In this review, we opted to follow the recommended name by Hamann et al. (2015) for adhesion GPCRs and the entry in diverse databanks: *Adgrl1*/ADGRL1, *Adgrl2*/ADGRL2 and *Adgrl3*/ADGRL3. The exception to that rule will be the *C. elegans* orthologs of ADGRLs, which will be abbreviated as *lat-1*/LAT-1 and *lat-2*/LAT-2 as those are the forms still employed in the adequate gene/protein repositories. Table 2 summarizes the nomenclature employed in this work regarding the latrophilins/ADGRLs.

**Table 2:** Nomenclature of the adhesion G protein-coupled receptors (ADGRLs).

Protein name	Protein abbreviation	Gene abbreviation	Synonymous notations	First use
***M. brevicolli***				
Adhesion G protein-coupled receptor L	ADGRL	*Adgrl*	MB7TM1	
***C. elegans***				
Adhesion G protein-coupled receptor L 1	LAT-1	*lat-1*		Willson et al., 2004
			B0457.1/B0286.2	Mee et al., 2004
Adhesion G protein-coupled receptor L 3	LAT-2	*lat-2*		Willson et al., 2004
***D. melanogaster***				
Adhesion G protein-coupled receptor L 1	ADGRL	*Cirl*	CIRL/latrophilin 1 (CL1)	Scholz et al., 2015
***D. rerio***				
Adhesion G protein-coupled receptor L 1	Adgrl1	*adgrl1*		
Adhesion G protein-coupled receptor L 2	Adgrl2	*adgrl2*		
Adhesion G protein-coupled receptor L 3	Adgrl3	*adgrl3*		
***G. gallus***				
Adhesion G protein-coupled receptor L 1	ADGRL1	*ADGRL1*		
Adhesion G protein-coupled receptor L 2	ADGRL2	*ADGRL2*	Latrophilin 2	Doyle et al., 2006
Adhesion G protein-coupled receptor L 3	ADGRL3	*ADGRL3*		
***M. musculus***				
Adhesion G protein-coupled receptor L 1	ADGRL1	*Adgrl1*	CIRL/latrophilin 1 (CL1)	Ichtchenko et al., 1998
Adhesion G protein-coupled receptor L 2	ADGRL2	*Adgrl2*	latrophilin-2 (Lphn2)	Anderson et al., 2017
Adhesion G protein-coupled receptor L 3	ADGRL3	*Adgrl3*	Latrophilin 3	Jackson et al., 2015
***R. norvegicus***				
Adhesion G protein-coupled receptor L 1	ADGRL1	*Adgrl1*	Latrophilin	Davletov et al., 1996
			Latrophilin (LPH1)	Lelianova et al., 1997
			Calcium-Independent Receptor of α-Latrotoxin (CIRL) 1	Krasnoperov et al., 1997
			CIRL/latrophilin 1 (CL1)	Sugita et al., 1998
Adhesion G protein-coupled receptor L 2	ADGRL2	*Adgrl2*	CIRL/latrophilin 2 (CL2)	Sugita et al., 1998
			Latrophilin 2 (LPH2)	Matsushita et al., 1999
Adhesion G protein-coupled receptor L 3	ADGRL3	*Adgrl3*	CIRL/latrophilin 3 (CL3)	Sugita et al., 1998
			Latrophilin 3 (LPH3)	Matsushita et al., 1999
***H. sapiens***				
Adhesion G protein-coupled receptor L 1	ADGRL1	*ADGRL1*	CIRL/latrophilin 1 (CL1)	Sugita et al., 1998
Adhesion G protein-coupled receptor L 2	ADGRL2	*ADGRL2*	CIRL/latrophilin 2 (CL2)	Sugita et al., 1998
Adhesion G protein-coupled receptor L 3	ADGRL3	*ADGRL3*	CIRL/latrophilin 3 (CL3)	Sugita et al., 1998


*The table presents a collection of synonymous presented in the literature for the latrophilins/ADGRLs found in different species and the corresponding protein and gene abbreviations*.

The ADGRLs are composed of multidomain regions possessing a rhamnose-binding lectin-like domain, an olfactomedin-like domain, and a hormone-binding domain that is similar to those of the CRF family of receptors (Matsushita et al., 1999). TEN2 binds and activates ADGRL1 with high affinity, while TCAP-2 binds to ADGRL2 inducing calcium release from intracellular stores (Silva et al., 2011). It was later shown that TEN4 also binds to ADGRL1, with a slightly lower affinity than TEN2, suggesting ADGRL1 is the cognate receptor of both TEN2 and TEN4 (Boucard et al., 2014). While the lectin-like domain of ADGRLs appear to be the most significant domain for teneurin binding and activation, it has been suggested that TCAP may interact with the hormone-binding domain (Woelfle et al., 2015).

## The Distribution of Teneurins/Tcap in Invertebrate Species

### *Caenorhabditis elegan*s

Although there is a single teneurin gene in *C. elegans* homologous to the two *D. melanogaster Ten* genes, this gene is controlled by two different promoters, what results in two different isoforms of TEN: a short version, TEN-S, and a long version, TEN-L. Promoter activity related to the TEN-L isoform was detected, during development, primarily on the derived cells of the EMS and C lineages, which give rise, respectively, to somatic tissues (endoderm, mesoderm, and stomodeum) and hypodermic and neural tissues (Drabikowski et al., 2005). Mörck et al. (2010) described a similar pattern at 150 min after fertilization, which will give rise to hypodermal cells by the end of gastrulation. In the somatic gonad, TEN-L is also active during gonadogenesis, what is reflected by its expression in z1 and z4 cells in the embryo. As early as 350 min, and consolidating at the 1.5-fold stage, expression of the long promoter can be detected in the pharynx, gut, and somatic gonad precursor cells, as well as in the aforementioned z1 and z4 cells (Drabikowski et al., 2005; Mörck et al., 2010).

During postembryonic development, a similar pattern was preserved. In the L1 larval stage, the pharynx, gut cells, hyp11 and somatic gonad precursors show TEN-L promoter activity, as well as z1 and z4 cells. At stage L4, fluorescence can be detected in some neurons of the nerve ring, as well as the anchor cell, vulva muscles and distal tip cells. In the adult hermaphrodite, staining is seen in the pharynx, selective nerve ring neurons, the vulva muscles, the distal tip cells and in coelomocytes. In the adult male, the same head structures are labeled in addition to the *vas deferens*, diagonal muscles and spicule sheath cells (Drabikowski et al., 2005). Mörck et al. (2010) also report strong expression of the long form of the transcript that persists in pharyngeal and intestinal cells and in several head neurons, including eight pharyngeal cells: the three marginal cells mc1, the three marginal cells mc3, and the neurons M2L and M2R.

The promoter associated to the TEN-S isoform, on the other hand, follows a distinct pattern of expression. During embryonic development, it is initially detected at 150 min after fertilization in anterior cells, although less abundantly than TEN-L. This signal can be traced to the descendants of lineage ABp. By 300 min after fertilization, the presence of TEN-S in posterior hypodermal cells becomes clear, in addition to ventral leading cells. Mörck et al. (2010) reports that the hypodermal expression then gradually fades away, with only head neurons being labeled by the end of embryogenesis. TEN-S post-embryonic activity was detected in specialized epithelial cells, such as the arcade cells of the anterior end and the excretory duct. The short promoter activity was also observed in a subset of neurons, consisting of CAN and HSN neurons, as well as lumbar and retrovesicular ganglion motorneurons and some nerve ring interneurons. In the adult, staining of TEN-S is limited to nerve ring, the ventral cord and a few cells in the tail, in addition to R8 and R9 ray neurons in the adult male (Drabikowski et al., 2005; Trzebiatowska et al., 2008).

To investigate the subcellular localization of different TEN isoforms in the *C. elegans*, Drabikowski et al. (2005) raised antibodies directed to the N- and C-terminus of TEN, with the C-terminal-raised antibody labeling only TEN-L, while the N-terminal antibody labels both isoforms in embryos. The C-terminal antibody labeled exclusively the plasma membrane, while the N-terminal antibody resulted in labeling of both the plasma membrane and a punctuate pattern of staining inside the nucleus. The authors suggested such staining pattern indicates the translocation of the N-terminal sequence of TEN as part of its signaling process in *C. elegans*.

### *Drosophila melanogaste*r

As stated before, two genes are found in Drosophila (*Ten-a* and *Ten-m*), encoding the transcripts TEN-a and TEN-m.

#### *Ten-*m

The expression of *Ten-m* starts around the blastoderm stage, 2 h after fertilization. Transcripts are found in the central area, outside the anterior and posterior poles, ubiquitously expressed on most of the embryo with exception of the dorsal side, where fewer transcripts are found. At the second half of germband elongation (5.5–7.5 h-old embryos), the diffuse expression of *Ten-m* is replaced by a quickly emerging pattern of stripes in mesodermal and ectodermal cells, that ends in a sharply defined pattern of 14 bands as germband elongation reaches its conclusion. During germ band retraction, the expression of *Ten-m* becomes more prominent at the dorsal margin of the germ band, and by the end of this stage transcripts are found in cardiac cells, lymph gland, posterior spiracles and in the tracheae. At this stage, expression also becomes clear in the nervous system, including the ventral cord and the supra-esophageal ganglia, with reminiscent patterned expression near the segmental furrows. At the time of hatching, transcripts are mainly confined to the ventral cord and to the brain (Baumgartner et al., 1994; Levine et al., 1994).

The synthesis of TEN-m follows a partially similar progression to its coding mRNA. Staining is located to the periphery of the cell, suggesting a plasma membrane localization that is common for several species. Immunoreactivity is detectable around the same time the transcripts are detected, encompassing the blastoderm stage. Its distribution overlaps with the mRNA, covering most of the central part of the embryo, but it’s absent of the anterior and posterior poles. A distinct small group of immunoreactive cells is found close to the anterior pole, which was identified as the anterior domain by Baumgartner et al. (1994). During gastrulation, a pattern of seven immunoreactive bands can be readily identified. This patterning occurs considerably sooner than that of the mRNA, suggesting TEN-m may be released and the bind to receptors that are already patterned in preparation of germ band elongation. As the stage progresses, the seven-band pattern becomes a fourteen-band pattern, and then immunoreactivity becomes almost exclusive to the mesoderm after a steep decline in ectodermal staining. As elongation advances, the banded pattern is reduced as the bands appear to fuse, and neuroblasts become stained at the anterior domain. At the time of germ band retraction, staining is visible on the tracheal system and in neurons along the midline. By the end of germ retraction, the pattern of immunoreactivity resembles once more that of the RNA, with staining on the ventral cord, cardiac cells and the lymph gland. During head involution, staining on axons of the ventral cord become prevalent, with a strict temporal pattern of synthesis (Baumgartner et al., 1994; Levine et al., 1994; Zheng et al., 2011).

During the larval stage, TEN-m immunoreactivity is found on axons and imaginal discs (Baumgartner et al., 1994). Novel synthesis is particularly evident on the *Drosophila* eye disc. TEN-m immunoreactivity in third instar larvae is evident at undifferentiated imaginal disc cells of the morphogenic furrow, a single cell in each maturing ommatidium, and a cluster of non-epithelial cells deep at the center of the eye disc. The appearance of one sharp staining centered point per ommatidia suggests its relation to the development of a photoreceptor cell with R7 identity. The third imaginal site of TEN-m synthesis can be described as a cluster of round adepithelial cells under the epithelial monolayer of the eye disc, which migrated from mesoderm germ into eye disc (Levine et al., 1997). *Ten-m* gene activity can also be detected on the morphogenetic furrow of the eye disc and in the brain optic lobes (Minet et al., 1999). It is likely, therefore, that the expression of *Ten-m* in both the developing eye and the optic lobe of the brain, and the presence of TEN-m immunoreactivity in the optic stalk, are indicative of a TEN-m function on the correct mapping between ommatidia and the visual lobe. As we will see, this function likely remained and was expanded in vertebrates. A distinct cluster of strong TEN-m staining cells are seen in the antennal, wing and leg discs, representing progenitors of a column of glial cells in which *Ten-m* is expressed. TEN-m immunoreactivity is very strong in the maxillary palps and rostral membrane that will give rise to the dorsal head capsule. Wing-disc staining of TEN-m is predominant in the wing blade, wing hinge and thoracic epidermis. Leg discs present rings of immunoreactivity, also characterized by a cluster of cells at the central core of the disc (Levine et al., 1997).

Information about the adult expression of *Ten-m* is limited to subjects immediately after eclosion. Staining is coherent with the disc staining, including strong staining in the three antennal segments, the maxillary palpus, the rostral membrane of the head capsule and derivatives of the clypeolabral and labial discs (Levine et al., 1997). Signals were detected in the brain and eyes but could not be explored in detail by the authors. It is noteworthy that the staining reported may be from residual beta-galactosidase expressed during pupation (Levine et al., 1997), so more studies in adult flies are necessary to confirm the expression of *Ten-m* in the adult animal.

#### *Ten*-a

The expression of *Ten-a* transcripts in the developing *D. melanogaster* is partially similar to that of *Ten-m*. Expression of the two start around the blastoderm stage, with widespread distribution. While *Ten-m* is not present on the anterior and posterior poles, *Ten-a* is uniformly distributed over the entire embryo. This uniform distribution persists through gastrulation, with a slightly more pronounced signal on the furrows. At the beginning of germ band elongation, signal becomes restricted to the ectoderm and mesoderm, when a pattern starts to appear at around 5 h of development. Although largely similar in timing, *Ten-a* transcription is better localized to the ectoderm, with only faint staining on the mesoderm, the opposite pattern of *Ten-m*. Clear signals can also be detected at the procephalic neuroblast region. As the germ band elongation progresses, the band pattern of *Ten-a* expression becomes clearer. By the time germ band retraction starts, it is possible to localize *Ten-a* to the ventral cord and the supraesophageal ganglion. At this stage, small labeled cells are seen near the segmental furrows, possibly representing sites of muscle attachment. During head involution, both the brain and the ventral cord show strong labeling, that will remain at the end of embryonic development and the three larval stages. *Ten-a* mRNA cannot be detected in the adult fly (Baumgartner and Chiquet-Ehrismann, 1993).

The described pattern of TEN-a immunoreactivity, however, is drastically different to that of TEN-m for the early period of embryogenesis. Fascetti and Baumgartner (2002) first detected TEN-a during germ band retraction, at stage 12, in neurons. Protein could be located to some cell bodies and on pioneering axons. By the end of germ band retraction, clear staining can be seen on the commissures of the ventral cord, especially on the posterior commissures. The hindgut is also labeled at this stage. During head involution, the pattern of TEN-a immunoreactivity becomes quite similar to TEN-m, including the brain, the ventral cord and structures of the antennomaxillary complex as the main sites of labeling. Low immunoreactivity is found in the CNS after differentiation (Fascetti and Baumgartner, 2002). Forty-eight hours after puparium formation, TEN-a can be detected in specific glomeruli. The subset of glomeruli synthesizing elevated TEN-a was distinct but partially overlapping with that synthesizing elevated TEN-m (Hong et al., 2012).

It is remarkable that *Ten-a* mRNA expression starts hours before the protein can be detected. A possible explanation for this observation is that, in early stages, *Ten-a* mRNA translation is silenced, or TEN-a is readily degraded after synthesis. This silencing is then selectively shut off in the neuronal lineage, allowing the protein to acquire a predominantly neuronal phenotype. Comparing the patterns of *Ten-a* and *Ten-m* expression and protein synthesis in *D. melanogaster* to that of *ten-1* in *C. elegans*, it is apparent that *D. melanogaster Ten-m* echoes the *ten-1* expression pattern of the common ancestor of *euarthropoda* and *nematoda*, while Ten-a differentiated its pattern of expression, becoming a late, predominantly neuronal-driven molecule in the insect lineage.

### *Ciona intestinal*is

No information regarding the teneurin distribution is available for the developing or adult *C. intestinalis*. The distribution of TCAP-1 has been investigated in the adult animal. Such scarcity of information certainly derives from the almost non-existent anatomical information about *C. intestinalis*. Since this information has been provided by Colacci et al. (2015), works describing the distribution of teneurin in these animals will be extremely informative.

#### Teneurin C-Terminal Associated Peptide (TCAP)

Immunoreactivity to TCAP in the adult *C. intestinalis* is found in the intestinal and sexual areas. In the testis, staining is found in putative Sertoli cells, outside of the tubules and in the epithelium of the sperm duct cells. In the ovary, labeling occurred in granulosa cell homologs and periovulatory cells, but not in the ovum. The subcellular localization of TCAP showed complex patterns depending on the region. In the intestine, labeling was detected both in the cytosol as in the periphery and along the plasma membrane. In the testis, most labeling in Sertoli cells was pericellular, with less staining inside those cells. In the ovary, on the other hand, labeling occurred primarily within the cytosol (Colacci et al., 2015). The distribution of TCAP mRNA was coherent, if broader, with that of the protein. RT-PCR results showed transcripts in the buccal siphon, central ganglion, branchial basket, testes, ovary, and stomach (D’Aquila et al., 2017).

## The Distribution of Teneurins/Tcap in Vertebrate Species

### *Danio* rerio

No morphological information is available for *D. rerio* Ten1, Ten2A, or Ten2B (Tucker et al., 2012).

#### *te*n3

Detection of *ten3* mRNA starts on the notochord and somites at tailbud stage, approximately 10 h after fertilization, what is coincident with the completion of the neural tube’s basic plan dorsal to the notochord. During segmentation, expression of *ten3* mRNA is not exclusively mesodermal anymore, as transcripts can be found in the developing nervous system. We will describe the mesodermal and ectodermal expression of *ten3* separately in this session (Mieda et al., 1999).

At 14 h after fertilization, *ten3* mRNA is strongly detected in the caudal forebrain, corresponding to the diencephalic area, while medium expression is found in the optic vesicles and midbrain. At this stage, a segmental expression of *ten3* mRNA is found on the rhombencephalon, with transcripts expressed in low levels in rhombomeres 3 and 5. By the end of the segmentation stage, additional expression of *ten3* mRNA is seen in the midbrain–hindbrain boundary. At 23 h post fertilization, *ten3* mRNA ceases to be detected in the hindbrain, it’s weakly detected in the anterior part of the midbrain–hindbrain boundary and becomes strongly expressed in the dorsal part of the tectal primordium, ventral part of the mesencephalon and caudal part of the diencephalon. At this stage, weak expression is found in the optic vesicles. By 36 h postfertilization, *ten3* mRNA expression is concentrated on the forebrain (Mieda et al., 1999). Further *ten3* mRNA expression has been described by Antinucci et al. (2013) in retinal cells. At 48 h postfertilization, expression is predominantly found in the ventral retina and medial portion of the stratum periventricular (tectal cells), while at 3 and 5 days postfertilization, *ten3* mRNA is diffusely expressed.

Regarding *ten3* mRNA mesodermic expression, at 14 hpf strong expression is found on the developing somites, while only low expression is seen on the notochord. At 17 hpf, a mediolateral gradient of expression develops, with the medial parts presenting weaker expression, and by 20 h *ten3* mRNA is detectable in the pharyngeal arches. By the end of segmentation, *ten3* mRNA is not detectable in the medial somites, and expression fully vanishes on somites by 36 hpf. At this stage, expression of *ten3* mRNA is observed in the pectoral fin buds and on pharyngeal arches (Mieda et al., 1999). No information is available about the distribution of *ten3* in the adult *D. rerio*.

#### *ten*4

In contrast to *ten3* mRNA, the expression of *ten4* mRNA is exclusively ectodermic in nature. By 10 hpf, *ten4* mRNA is expressed along the anterior margin of the neural plate, and by 14 h postfertilization the brain is the main site of expression. At this stage, the pattern of *ten4* mRNA is partially complimentary to *ten3* mRNA, with transcripts found on the forebrain, including the optic vesicles (albeit in lower levels than *ten3* mRNA) and the rostral diencephalon, the mesencephalon, and in rhombomere 5 and 6 of the rhombencephalon. On 20 hpf, the segmental pattern of *ten4* mRNA becomes stronger, with expression found in the rostral diencephalon, the mesencephalon, the mesencephalon-hindbrain boundary and rhombomere 2 (previously clear of *ten4* mRNA expression). Expression in rhombomeres 5 and 6 persist and increase in intensity. Finally, transcripts are also found in the anterior spinal cord, with additional weak expression of *ten4* mRNA is found in individual neurons of the caudal spinal cord. At 23 hpf the distribution of *ten4* mRNA strongly diverges of that of *ten3* mRNA, with dorsal and ventral bands of expression in the rostral diencephalon, weak expression on the caudal diencephalon, mesencephalic expression on the ventral part of the tectal primordium, strong expression in the caudal mesencephalic-hindbrain boundary, very strong expression on rhombomeres 2, 5, and 6 and in the anterior spinal cord. Widespread expression is found in the brain at 36 hpf (Mieda et al., 1999). No information is available about the distribution of *ten-4* in the adult *D. rerio*.

Comparing the expression of teneurins in *D. rerio* to the distribution of *C. elegans ten-1* and *D. melanogaster Ten-a* and *Ten-m*, a pattern can be distinguished. *D. rerio ten3* has a similar pattern of expression to the *C. elegans* teneurin and the *D. melanogaster Ten-m*, as it is found in both mesodermal and ectodermal tissue early during embryogenesis and then becomes prevalent in neural cells during axiogenesis and connection formation. On the other hand, *D. rerio ten4* mRNA carries semblance to *D. melanogaster Ten-a*, being utilized later during embryogenesis by the nervous system in a pattern partially complementary to that of *ten-3 mRNA*. As commented before, however, it is likely that a duplication event happened specifically on the insect lineage to generate *Ten-a* and *Ten-m*, while the four vertebrate ten genes result from two other events of duplication specific to the early vertebrate lineage. The similarities between *ten3* and *Ten-m* and *ten4* and *Ten-a*, therefore, must result from evolutionary convergence, rather than homology itself. This is underscored by the fact that the mechanism that regulates the nervous system-specific paralog in each species is different: while in *D. melanogaster Ten-a* is expressed early but the protein only appears late, be it by RNA silencing or protein degradation, in *D. rerio ten4* mRNA will only be detectable once the nervous system begins to differentiate. We cannot exclude, however, the possibility that the presence of two different promoters for the teneurin gene early in evolution may have facilitated the differentiation of functions once the gene was duplicated, what may have contributed to the retention of multiple paralogs and may have guided how the new patterns of expression/synthesis emerged.

Although *ten3* and *ten4* are equidistant to the *C. intestinalis* teneurin (Tucker et al., 2012), it is likely that, from a morphofunctional perspective, *ten3* represents a more conserved expression pattern when compared to early forms of teneurin. More studies in *D. rerio* are necessary to establish if the complementary pattern of expression between *ten3* and *ten4* developed before or after the genic events that resulted in the creation of *ten1*, *ten2A*, and *ten2B*.

### *Gallus gallu*s

#### *TEN*1

Unfortunately, expression analyses of *TEN1* mRNA are not available for the early embryogenesis of chicken. By day 5, transcripts are detected on the embryo head, but not the trunk. On day 7, a hybridization signal can be found only in the developing nervous system. By days 14 and 17, strong signals are found in the tectofugal elements of the visual system, such as retinal ganglion cells, the *stratum griseum centrale* of the optic tectum, and the rotund nucleus of diencephalon. Further signals are also detected in the inner nuclear layer of the retina and in other layers of the optic tectum. Areas linked to olfactory sensing and processing are also stained, such as the mitral cells of the olfactory bulb and neurons from the hippocampus and piriform cortex. In the hindbrain, transcripts were found in the nucleus laminaris, nucleus magnocellularis and throughout the cerebellum. TEN1 immunoreactivity is largely compatible with the aforementioned distribution, but extends to some regions connected with *TEN1* mRNA synthesizing areas, such as the glomerular layer of the olfactory bulb (which accommodates the dendrites of mitral cells) and the outer portion of the inner nuclear layer of the retina (Minet et al., 1999; Rubin et al., 1999; Kenzelmann et al., 2008). Information about *TEN1* mRNA in the adult chicken is limited. Minet et al. (1999) detected signals for *TEN1* mRNA in northern blot experiments of adult chicken brain extracts. No signal was detected in adult kidney, heart or liver.

#### *TEN*2

The expression of *TEN2* and TEN2 immunoreactivity are both found in neuroectodermal and mesodermal tissues during development, and that is why these two sites of expression/synthesis will be addressed separately.

Trunk expression of *TEN2* mRNA can be detected as early as 3 days after incubation, during late somitogenesis stage. At this stage, transcripts can be found in branchial arches, heart, somites, craniofacial mesenchyme and the apical ectodermal ridge of developing buds. After 5 days of incubation, transcripts are seen in the wing and hindlimb, the trunk, maxillary and mandibular processes and the head mesenchyme. At 7 days of development, the signals disappear (Tucker et al., 2001). Immunohistochemistry for TEN2 resulted in agreeable results to those of *in situ* hybridization, including the cranial mesenchyme, branchial arches, the developing somites, and the apical ectodermal ridge. The only exception was the notochord, which was stained with the antibody but did not show signs of *TEN2* mRNA expression. The investigation of TEN2 immunoreactivity outside the nervous system also gave clues about the subcellular localization of TEN2 in the avian model, as the punctuate outlining-pattern of staining resulting from immunohistochemistry is expected from a membrane-anchored protein (Tucker et al., 2001).

In the nervous system, *TEN2* mRNA is first detected at 4 days after incubation. In 7-days old embryos, transcripts are found in the retina, telencephalon, diencephalon and the optic tectum, in a pattern that mostly overlaps with that of *TEN1* mRNA, except in the diencephalon, where *TEN2* mRNA is found on the anterior thalamus, while *TEN1* mRNA is prevalent on the dorsal thalamus. As was the case of *TEN1*, most of *TEN2* expression was concentrated on members of the tectofugal system. In day 10 after incubation, strong hybridization signals are found in the forebrain, particularly on the hippocampus and in the visual Wulst, and by day 12 the retinal cell ganglion is clearly labeled. By 14 days after incubation, *TEN2* mRNA expression concentrates on the *stratum griseum periventriculare* of the optic tectum, mostly separated from *TEN1* mRNA in the *stratum griseum centrale*. Additional signals are found on the lateral geniculate nucleus (Rubin et al., 1999; Rubin et al., 2002).

Immunoreactivity to TEN2 was similar to gene expression, with some additional areas of staining that were not previously found. On day 7 post incubation, labeling can be seen in the retinal nerve fiber layer. Labeling has expanded to other elements of the visual system by day 11, including the inner plexiform layer, the optic nerve and the optic tectum. As was the case with the messenger RNA, by day 17/18 post incubation the synthesis of TEN2 becomes more widespread. On the visual system, immunoreactivity is found in the inner plexiform layer of the retina, visual Wulst, the ventral geniculate nucleus, pretectal nuclei, *stratum griseum periventriculare* and *centrale*. Some of these areas show remarkable separation between TEN1 and TEN2, such as the retina (TEN2 is found in *laminae* 1 and 3, while TEN1 is found in *laminae* 2 and 5) and the optic tectum (while TEN1 appears to be actively synthesized by *stratum griseum centrale* cells, TEN2 is found in a punctiform manner that suggests its synthesis by cells in other areas that are synapsing at the *stratum griseum centrale*). Other areas that include TEN2 immunoreactivity are the olfactory bulb, piriform cortex, hippocampus, septal nuclei, and cerebellum. In several cases, TEN2 immunoreactivity was found associated to the presence of a basement membrane (Tucker et al., 2001; Kenzelmann et al., 2008). In the adult, *TEN2* mRNA transcripts are detected by northern blot and RT-PCR in the adult brain, but no signals are found in the heart or liver (Rubin et al., 1999).

Looking at the distribution of *TEN1* and *TEN2* in the *Gallus* brain, we see several of the morphofunctional aspects exhibited as early as in *C. elegans*, as well as the more complex patterned expression between *TEN1* and *TEN2* that will be characteristic of integrative areas of the amniote brain. In several ways, the expression and synthesis of *TEN2* is evocative of the *C. elegans* teneurin, the *D. melanogaster Ten-m*, and the *D. rerio ten3*, including its expression in both non-neural and neural tissues, the timing of expression, and its association to the basement membrane. On the other hand, *TEN1* is evocative of the Drosophila *Ten-a* and the *D. rerio ten4*, with a predominantly neural expression that occurs in tandem with that of the other paralog in regions of great connectivity, such as the visual pathway and olfactory areas.

#### *TEN*3

No information is available for the *G. gallus TEN3* expression or TEN3 synthesis.

#### *TEN*4

The largest body of evidence available points to a peripheral expression of *TEN4* mRNA in non-neuronal tissue of the developing chicken. Transcripts are first detected 3.5 days after incubation in zones of polarizing activity (ZPA), branchial arches, cells lining the intersomitic clefts and in the cranial mesenchyme. As development progresses, increased signals are detected in the mesenchyme, including the mesenchyme dorsal to the dorsomedial lip of the somites, the cranial mesenchyme and the mesenchyme of the first, second and third brachial arches. By 5 days of development, however, most of the signal is found in the developing limbs, including additional patches of expression (outside the zones of polarizing activity) in the leg and wing buds (Tucker et al., 2000). Unfortunately, little has been described about the distribution of *TEN4* mRNA in the developing nervous system of the chicken, except for the observations of Tucker et al. (2000) that transcripts can be seen in the midbrain–hindbrain junction and in the diencephalon.

### *Macropus eugeni*i

#### *TEN*3

Carr et al. (2013, 2014) investigated the post-natal development of ipsilateral retinogeniculate projections in the wallaby marsupial (*Macropus eugenii*). In these animals, *TEN3* mRNA can be found in the retina, with labeling concentrated on the retinal ganglion cell layer and in the superior colliculus, where labeling is found in the superficial retinorecipient layers at all ages examined, from P12 to P99, and in the adult. Additional labeling was found in the dorsal part of the lateral geniculate nucleus of P12–P71 animals, but older animals were not examined. The main difference between developing and adult animals was the existence of dorsoventral (retina) and mediolateral (superior colliculus) gradients in the young subjects, which disappeared in the adult.

### *Mus musculu*s

#### *Ten*1

The earliest reported expression of *Ten1* mRNA in the developing mouse is at E13.5. By E15.5, transcripts can be detected in subplate and cortical plate in a rostral-low/caudal-high, dorsomedial-low/ventrolateral-high gradient. *Ten1* mRNA is also detected in the dorsal thalamus at this stage, including the ventroposterior nucleus, posterior complex and lateral geniculate nucleus, dorsal part. By E18.5 the gradient is reversed, with a rostral-high/caudal-low pattern. At P2, the overall pattern of expression stays the same, with *Ten1* mRNA found in layer 4 and subplate in the cortex, while low signals are found in layers 5 and 6. Low expression also remains in the thalamic ventroposterior nucleus, and strong expression is found in the thalamic reticular nucleus. By P7, additional expression is seen on CA1, CA3 and dentate gyrus of the hippocampal formation (Li et al., 2006). The distribution of *Ten1* mRNA is likely to be more widespread than that, as Zhou et al. (2003) describe hybridization signals for *Ten1* mRNA in the midbrain, hindbrain, spinal cord, and trigeminal ganglion. Furthermore, despite not described by the authors, it is clear in the work of Li et al. (2006) that other areas of the brain are stained in the developing brain, including midline nuclei of the thalamus, the amygdaloid complex and discreet nuclei of the hypothalamus. No data is available on non-neuronal expression of *Ten1* mRNA in the mouse.

In the adult mouse, Oohashi et al. (1999) report the presence of *Ten1* mRNA transcripts in the adult brain, in addition to faint signals in the kidney, testis, and thymus. In 6-week old animals, prominent *Ten1* mRNA hybridization signals are found in the *stratum pyramidale* of the CA2 subfield of the hippocampus proper and in the granular layer of the dentate gyrus, while weak expression is found in the CA1 and CA3 subfields. TEN1 immunoreactivity in the hippocampus at this stage was only partially similar, with stronger staining in the stratum lucidum of the CA3 region and weaker staining in CA1 and dentate gyrus. This mismatch between expression and immunoreactivity likely reflects the intra-hippocampal connectivity, with immunoreactivity found in the axons of neurons that express *Ten1* mRNA (Oohashi et al., 1999; Zhou et al., 2003). The cerebellum also contained *Ten1* mRNA transcripts, which were found in the granular layer. The protein was found in the molecular and granular layers, in addition to cerebellar nuclei (Oohashi et al., 1999; Zhou et al., 2003). Additional hybridization/protein is found in the cerebral cortex (layers 2–6) and thalamus, and protein has been found in the brainstem and retina (Oohashi et al., 1999; Zhou et al., 2003). Outside the brain, TEN1 is found in the lung, kidney and in the testes. In the latter, immunofluorescence signals are detected in the *tunica propria* of the seminiferous tubule and the surrounding interstitial cells (Oohashi et al., 1999; Chand et al., 2014).

#### TCAP-1

The presence of TCAP-1 immunoreactivity was examined in adult BALB/c mice using an antibody directed to its sequence. TCAP-1 immunoreactivity is observed in the pyramidal layer and *stratum oriens* of the CA1 subfield; pyramidal layer, *stratum lucidum* and *stratum radiatum* of the CA2/CA3 subfields, and additional weaker staining in the granular layer of the dentate gyrus (Chand et al., 2013). Outside the nervous system, TCAP-1 immunoreactivity is found in germ cells and spermatocytes adjacent to the basement membrane (Chand et al., 2014).

#### *Ten*2

Expression of *Ten2* mRNA in the developing mouse starts around E10.5, when hybridization signals are positive in the forebrain, rostral and central midbrain, the outer linings of the optic cup and in the auditory vesicle. At E12.5, expression becomes more widespread and can be detected in the caudal forebrain, very strongly on the midbrain, strongly in the hindbrain and weakly on the spinal cord. By E15.5, transcripts are more clearly located to the roof of the midbrain, in addition to the hindbrain and the nasal cavity. In the telencephalon, *Ten2* mRNA transcripts are distributed in a rostral-low/caudal-high within the cortical plate. Expression is also found in the diencephalon, including the centrolateral nucleus, dorsal part of the laternal geniculate nucleus posterior complex and ventral part of the medial geniculate nucleus, ventral thalamus, and discreet nuclei of the hypothalamus. During development, the cortical gradient of *Ten2* mRNA remains the same. By P2, *Ten2* is widely expressed in the cortex but the strongest signal is found in layer 5. A new site of expression after birth is the hippocampal formation, with high expression in CA1 when compared to the weaker signals of CA3 and DG. By P7, the different levels of expression in the hippocampus equalize (Zhou et al., 2003; Li et al., 2006).

Immunoreactivity data for TEN2 during mouse development is available in the investigation of Young et al. (2013) of the developing visual pathway. At E14, immunoreactivity is found in the retinal ganglion cell layer within the central retina, while by E16 the dorsoventral axis has become uniform. Synthesis of *TEN2* is ample on axons of the retinofugal pathway, including the optic nerve, optic tract, and optic chiasm. Targets of the retina are also immunoreactive to TEN2, including the superior colliculus and the dorsal part of the lateral geniculate nucleus, and such synthesis was observed from E16 until P7. By P7, immunostaining can be identified on layers 4 and 5 of the primary visual cortex (Young et al., 2013).

In 6-week old adults, *Ten2* mRNA transcripts are found in the brain, kidney, and testis (Oohashi et al., 1999; Ben-Zur et al., 2000). In the brain, *Ten2* mRNA was thoroughly expressed in the pyramidal and granular layers, with immunoreactivity found in the *stratum oriens*, *stratum radiatum*, and *stratum lacunosum moleculare* of the CA1/CA2 subfields (Zhou et al., 2003). In the cerebellum, *Ten2* mRNA is found in the molecular layer and Purkinje’s cell layer. Immunoreactivity, on the other hand, is found in the molecular and granular layers. Finally, expression and synthesis of *Ten2*/TEN2 are found on layers 2–6 in the cerebral cortex (Zhou et al., 2003).

#### *Ten*3

As more information is available about *Ten3* mRNA expression in non-neuronal tissues, this distribution will be discussed separately from that in neuronal tissue. At E7.5, high levels of *Ten3* mRNA are found in the notochord. Two days later, *Ten3* mRNA is detected on anterior somites and limb buds. By E10.5, expression starts to be detected in the first, second, and third branchial arches. At E12.5, expression of *Ten3* mRNA is found in the facial mesenchyme and in the head meninges, and at E16.5 transcripts are seen in the mesentery of the gut and the urogenital system. Finally, at E18.5, expression is seen in the dermis, developing limbs and in the outer layers of the periosteum and muscle epimysium (Ben-Zur et al., 2000; Zhou et al., 2003).

In neural tissue, *Ten3* mRNA is detected as early as E7.5 in the neural plate, with higher expression levels in the neural folds. At E8.5, transcripts are found in the caudal forebrain, dorsal midbrain region and in the otic vesicle. A day later, *Ten3* mRNA expression can be seen in the telencephalon, diencephalon, dorsal midbrain and in the otic vesicle (Zhou et al., 2003). At E10, expression has also been reported in the Rathke’s pouch (Ben-Zur et al., 2000). By E12.5, the optic tectum becomes the most preeminent site of *Ten3* mRNA expression. Hybridization signals are also seen in all layers of the neocortex, in the hippocampus, and in the thalamus. At this developmental stage, high levels of transcription are seen in the optic recess of the diencephalon, in the optic stalk, in lens cells and in the corneal ectoderm. Restricted signals are detectable in the pons and the rostral medulla at this stage, as well as in the dorsal horn of the spinal cord. At E15.5, cortical plate expression of *Ten3* mRNA follows a low-rostral/high-caudal gradient. In the dorsal thalamus, transcripts are detected in the centrolateral nucleus, dorsal part of the lateral geniculate nucleus, ventral part of the medial geniculate nucleus and posterior complex. At E16.5, the retinal expression of *Ten3* mRNA shifts to the inner neuroblastic layer, with a high-ventral/low-dorsal gradient that will remain into the first postnatal week. In the superior colliculus, a high-medial/low-lateral gradient of *Ten3* mRNA is observed in the superficial retinorecipient layers. Outside the central nervous system, low levels of expression were found in dorsal root ganglia while higher levels were found in trigeminal ganglia (Ben-Zur et al., 2000; Zhou et al., 2003; Li et al., 2006; Leamey et al., 2007a; Dharmaratne et al., 2012).

Shortly after birth, on P0, *Ten3* mRNA transcripts are found on layer 5 of the developing virtual cortex, while the protein is found in the same region as well as in axons projecting from this area. P2 expression of *Ten3* mRNA in the cortex is detectable in layers 5 and 6, with the somatosensory cortex also displaying labeling in layer 4. In the hippocampus, staining is seen in CA1 exclusively. Thalamic expression has increased and includes the centrolateral nucleus, ventral posterior nucleus, lateral posterior nucleus, posterior complex, lateral geniculate nucleus and reticular thalamic nucleus. At P3, *Ten3* expression can also be detected in the striatum, with a stronger labeling in the dorsal striatum following the characteristic striatal organization in patches. This pattern is conserved by P7 (Li et al., 2006; Leamey et al., 2007b; Tran et al., 2015). At P10, TEN3 immunolabeling is seen in the proximal CA1, distal subiculum and medial entorhinal cortex. TEN3 was most prominent in synaptic layers, including the *stratum lacunosum-moleculare* of CA1 and the molecular layer of the subiculum, what is consistent with TEN3 being present in the synaptic cleft. TEN3 was also present in axons, dendrites and cell bodies (Berns et al., 2018).

In 6-week old adults, *Ten3* mRNA transcripts are found in the brain, liver and testes (Oohashi et al., 1999; Ben-Zur et al., 2000). In the hippocampus, the expression of *Ten3* was relatively lower when compared to other teneurin paralogs, with transcripts found in the CA2 subfield and weakly in CA1. TEN3 immunoreactivity was broadly found in the cerebellum, including the molecular, granular and Purkinje’s cells layers and the white matter. Hybridization signals for *Ten3* were found in layers 2–6 of the cerebral cortex (Zhou et al., 2003).

#### *Te*n4

Outside the nervous system, *Ten4* mRNA transcripts are first detected in the mesentery of the gut, as early as E7.5 (Ben-Zur et al., 2000). Expression is then detected in the posterior somites and the tail bud at E9.5, and by E10.5 *Ten4* mRNA was also observed in the periocular area and in the first, second and third branchial arches (Zhou et al., 2003). At E12.5, ample expression of *Ten4* mRNA is detected in the facial mesenchyme, nasal epithelium, trachea, mesentery of the gut and urogenital system. By E18.5, *Ten4* mRNA is expressed by the epidermis of the skin and the developing joints between bones in the limbs and in adipose tissue, but this expression subsides as birth approaches (Ben-Zur et al., 2000). It is noteworthy that Lossie et al. (2005) found a radically different pattern of *Ten4* expression in the developing mouse, with transcripts found in the epiblast by E6.5, in the mesoderm of the developing embryo by E7.5, and expression exclusive to the tail bud and limbs in E11.5 embryos. It is likely that the probe used by Lossie et al. (2005) detected a splicing variant of *Ten4* that was different from that of Ben-Zur et al. (2000) and Zhou et al. (2003). The existence of multiple splicing variants with specific patterns and timings of appearance in the developing embryo considerably increases the complexity of studying the morphology of the teneurins and represents a challenge to be overcome in future studies.

In the nervous tissue, *Ten4* mRNA is detected at E7.5 in the neural plate. At E8.5, transcripts are detected in the caudal forebrain and the rostral midbrain region. Considerable expansion in expression occurs in E9.5, with *Ten4* mRNA found in the alar and basal regions of the caudal diencephalon and in the midbrain–hindbrain boundary, including the caudal alar mesencephalon and the basal rostral rhombencephalon. At this stage, weak expression of *Ten4* mRNA starts to be detected in the cortex. At 12.5E, low levels of expression are found in all layers of the cerebral cortex, with higher levels found in the mantle layer, and in the hippocampus. The diencephalic staining at this stage becomes sharper, with signals localized to the medial thalamus, the mammillary bodies, and the optic recess. The inferior colliculus and the optic tectum are also labeled at this stage, as well as the saccule. At E15.5, *Ten1* and *Ten4* show an overlapping expression in the cortex, with *Ten4* mRNA found in a low-rostral/high-caudal gradient in both differentiating cells in the cortical plate and proliferating cells in the ventricular zone. Thalamic expression can be pinpointed to the dorsal lateral geniculate nucleus, ventral medial geniculate nucleus and posterior complex. Finally, at E16.5, *Ten4* mRNA expression is seen in the inner neuroblastic layer of the retina. In the periphery, *Ten4* mRNA is found in high levels in the dorsal root ganglia and in lower levels in the trigeminal ganglia. After birth, the low-rostral/high-caudal gradient of *Ten4* mRNA expression in the cortex is maintained at least in P2 and P7, as well as in the thalamus and in the CA1 field of the hippocampus (Wang et al., 1998; Ben-Zur et al., 2000; Zhou et al., 2003; Li et al., 2006).

In 6-week old adults, *Ten4* mRNA transcripts are found in the brain, liver and testes (Oohashi et al., 1999; Ben-Zur et al., 2000). In the hippocampus, expression of *Ten4* is weakly found in the granular layer of the dentate gyrus and in the *stratum lacunosum moleculare*, as well as in the entire *stratum pyramidale*. Immunoreactivity to TEN4, on the other hand, was prominent in both molecular layers of the dentate gyrus, *stratum lacunosum pyramidale* and *stratum oriens* of the CA3 subfield. In the cerebellum, hybridization signals for *Ten4* mRNA were found in the Purkinje’s cells zone and in the white matter, while immunoreactivity was strongly found in the granular layer and only weakly observed in the molecular layer and white matter. Hybridization signals for *Ten4* mRNA were found throughout layers 2–6 of the cerebral cortex and in the thalamus (Zhou et al., 2003).

### *Rattus norvegicu*s

#### *Ten*1

The expression of *Ten1* mRNA in the nervous system starts weakly at E16 and increases in intensity from E17 forward. In the E17 cerebral cortex, *Ten1* mRNA is found in a low-rostral/high-caudal gradient, with signals mostly located to pyramidal cells in the cortical plate and subplate. In the thalamus, the anterior and intermediate parts were more strongly labeled than the posterior area. The midbrain, the hypothalamus, cerebellum, pons, medulla and spinal cord were also positive for signals. Proliferating zones were free of staining. The main olfactory bulb shows transcripts in the external, middle and internal tufted cells, and the accessory olfactory bulb is staining in its caudal-most area. By E19, the output cell layer of the accessory olfactory bulb is strongly stained, and the main olfactory bulb has uniform expression throughout the external plexiform layer. At this stage, staining intensity increases in the thalamus and the septum, and signals start to be detected in the subicular area, hippocampus, and dentate gyrus. By E21, signal is strong in the cortex, thalamus, septum and midbrain. At this stage, weak signals are detected in the rhinencephalon. After birth, several sites of *Ten1* mRNA waned, including the cortex, the thalamus, the septum, the midbrain, the hypothalamus and the rhinencephalon. Staining in tufted cells was significantly decreased by P1 and vanished completely by P5, but granule cells started expressing *Ten1* mRNA at P3. In the accessory olfactory bulb, *Ten1* mRNA expression increased by P3 but vanished by P5. By P30, the expression of *Ten1* mRNA increased in the granule cells of the dentate gyrus and in pyramidal cells of the hippocampus proper (Otaki and Firestein, 1999).

#### TCAP-1

**FIGURE 4:**
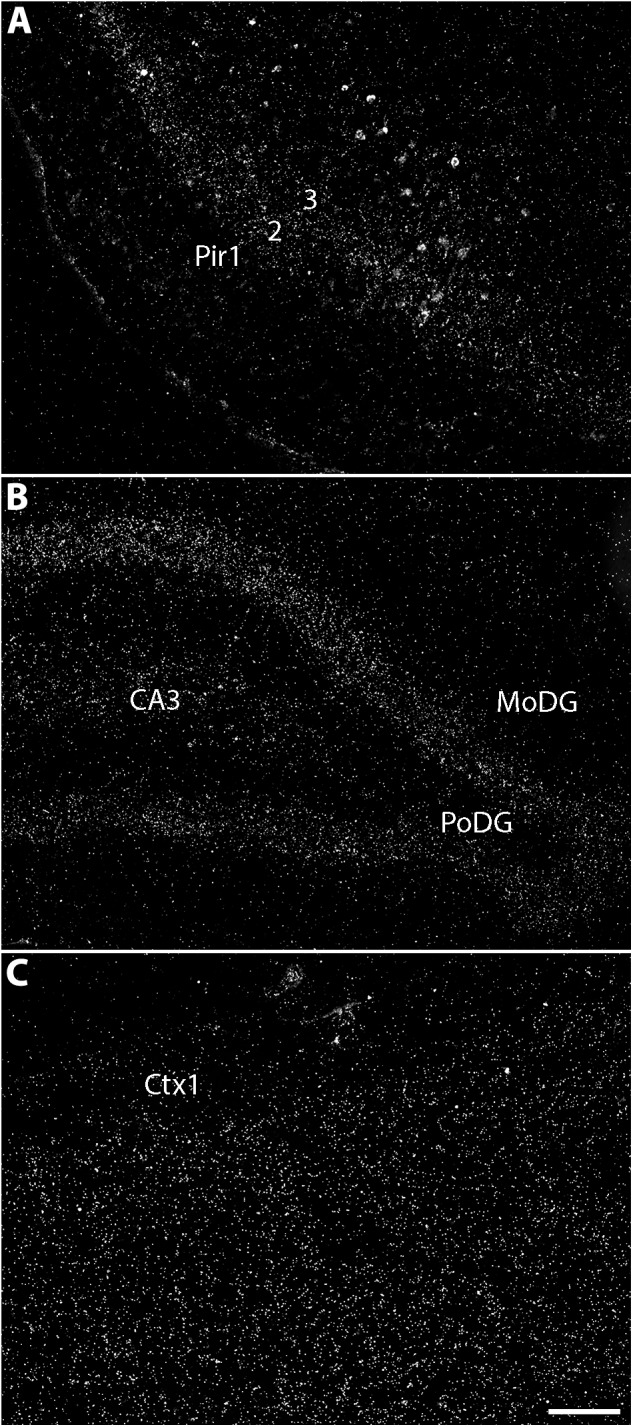
The telencephalic expression of TCAP-coding mRNA in the rat. Darkfield photomicrographs of coronal male rat brain slices that underwent *in situ* hybridization for the localization of TCAP-coding mRNA. It is noteworthy that TCAP-coding mRNA staining pattern is diffuse and seldom aggregate in cells, as seen for other markers. **(A)** The piriform cortex has one of the highest concentrations of mRNA, particularly in layer 2 and, to a lesser extent, in layer 3. **(B)** The pyramidal layer of the dentate gyrus also contains a high density of *in situ* hybridization labeling, while some degree of staining can also be seen in CA3 of the hippocampus proper. **(C)** The somatosensorial cortex has ample expression of TCAP-coding mRNA, with low expression in layer 1 and high, uniform expression on layers 5 through 6. CA3, cornus ammonis 3 of the hippocampus proper; Ctx, somatosensorial cortex, layer 1; MoDG, molecular layer of the dentate gyrus; PoDG, polymorphic layer of the dentate gyrus; Pir1, piriform cortex, layer 1; Pir2, piriform cortex, layer 2; Pir3, piriform cortex, layer 3. Scale bar: 100 μm. Based on the data published by Wang et al. (2005).

The distribution of the C-terminal exon of *Ten1* was investigated as a proxy for TCAP-1 expression in the adult rat brain by Wang et al. (2005). Several brain areas were positive for TCAP-1-corresponding transcripts, including allocortical areas (olfactory bulb, piriform cortex, hippocampus proper and dentate gyrus), the central and basolateral nuclei of the amygdala, the ventromedial nucleus of the hypothalamus, the subthalamic nucleus, the vagus and hypoglossal nuclei, and the Purkinje cell layer of the cerebellum. Figure 4 shows the main prosencephalic regions presenting TCAP-1 immunoreactivity.

#### *Ten*2

The only available data about TEN2 synthesis and *Ten2* expression in the rat is in the developing teeth. Ectomesenchymal cells of the dental papilla subjacent to the internal enamel epithelium layer are immunoreactive to TEN2 starting at bell stage, by E20. Later at this stage there is an increase in labeling intensity in the odontoblast cell layer adjacent to pre-ameloblasts. In postpartum animals (P0-P7), TEN2 immunoreactivity is still detected in odontoblasts during crown formation. In mature teeth, TEN2 is diffusely distributed in the cell bodies and processes of odontoblasts of the coronal and radicular pulps. The expression patterns of *Ten2* mRNA and that of exon 28 of *Ten2* (corresponding to the TCAP-2 region of the protein) were coherent with that of the protein (Torres-da-Silva et al., 2017).

### *Homo sapien*s

#### *TEN*2

Torres-da-Silva et al. (2017) investigated immunoreactivity to TEN2 in the dental pulp of adult humans. Coronal dental pulp fragments evidenced a uniform distribution of TEN2-immunoreactivity only in odontoblasts. The authors report that, sometimes, the initial segment of the odontoblastic process was preserved, showing TEN2-immunoreactivity, similar to rat odontoblasts. RT-PCR analysis confirmed expression of *TEN2* and that of exon 29 of *TEN2* (corresponding to the TCAP-2 region of the protein) in human coronal pulp samples. Tews et al. (2014, 2017) detected the expression of *TEN2* mRNA in adipocyte precursor cells, with higher expression on white cell precursors when compared to brown cell precursors.

#### *TEN*4

Graumann et al. (2017) investigated *TEN4* expression in the ovary of normal and tumoral patients. The expression of *TEN4* mRNA was detected by PCR in the normal ovary.

## The Distribution of Adgrls

### *Caenorhabditis elegan*s

The expression of *lat-1* reporter begins on the zygote and is more evident in the AB lineage. In early development, *lat-1* is broadly expressed in a stripped pattern visible in epidermal and pharyngeal precursors during dorsal intercalation. In larval and adult stages, *lat-1* is expressed in the pharynx, the nervous system, the gonad and the vulva (Langenhan et al., 2009). The expression of *lat-2* is generally imbricated with *lat-1* in the pharyngeal primordium and it is restricted to the pharynx and the excretory cell in later stages (Langenhan et al., 2009).

### *Drosophila melanogaste*r

The main sites of *Cirl* expression in *D. melanogaster* are the larval CNS and the brain. Other enriched areas with *Cirl* mRNA expression are the thoracoabdominal ganglion, the salivary gland, the head and the eyes^1^ (van der Voet et al., 2016). *Cirl* is expressed in several peripheral sensory neurons of the *D. melanogaster* larva, including type I and type II neurons. Expression of this gene was most prominent in larval pentascolopidial chordotonal organs (Scholz et al., 2015).

### *Gallus gallu*s

Doyle et al. (2006) investigated the presence of *ADGRL2* on heart formation in stage 5, 16 and 21 of chicken embryos. By using *in situ* hybridization, the author observed *ADGRL2* expression in the mesoderm leaving the primitive streak of the stage 5 embryo and also a weak labeling in the epiblast. In the stage 16, *ADGRL2* expression was detected in forming somites, notochord, nephric ducts and the cardiac endothelium. At stage 21, immunostaining was observed in both the endothelium and the mesenchyme of the cardiac cushions as well as the muscle.

### *Mus musculu*s

*In situ* hybridization of P7 mouse brain slices revealed *Adgrl1* expression in layer 5 of the cerebral cortex, the anterodorsal and anteroventral nuclei of the thalamus and the internal and external glanular layers of the cerebellum. Signals were also detected in the subiculum and the CA1 region of the hippocampus. Immunostaining in P14 mice confirmed the presence of ADGRL1 in layer 5 of the cerebral cortex and in the anterodorsal and anteroventral nuclei of the thalamus (Zuko et al., 2016).

### *Rattus norvegicu*s

Northern blot analysis of different adult rat tissues analyzed revealed the presence of *Adgrl1* exclusively in the brain, and not in liver, heart, lung, kidney, spleen, skeletal muscle, and duodenum. In the brain, Western Blot of different brain areas revealed that ADGRL1 was most preeminent in the striatum, somewhat lower in cortex and hippocampus, and much less in the cerebellum (Krasnoperov et al., 1997).

Matsushita et al. (1999) investigated the distribution of *Adgrl1* mRNA in northern blots of RNA isolated from different adult rat tissues. *Adgrl1* mRNA was almost exclusively brain-specific, with very low levels of *Adgrl1* detected in kidney, lung and spleen. The concentration of *Adgrl1* mRNA in the brain is leastways 50-fold higher in the brain than in any other tissue. No ADGRL1 was detected in liver samples by Western Blotting.

Matsushita et al. (1999) found that *Adgrl2* mRNA expression was prevalent in liver, lung and brain tissues, but were found to variable extent in all tissue tested (heart, spleen, muscle, and kidney). Disagreeing with the RNA expression, however, Western Blot experiments performed by Matsushita et al. (1999) could not find the ADGRL2 protein in the brain. Matsushita et al. (1999) also investigated the distribution of *Adgrl3* mRNA in different adult rat tissues. As is the case of *Adgrl1*, *Adgrl3* mRNA was found mostly in the brain and in lower amounts in the lung and spleen.

### *Homo sapien*s

According to Sugita et al. (1998), *ADGRL1* mRNA was largely enriched in human brain samples, but longer exposure times revealed *ADGRL1* expression in several other tissues, including the placenta, lung, kidney and pancreas. PCR confirmed that *ADGRL1* is expressed in fibroblasts. The authors suggest that the failure to observe *ADGRL1* expression outside the CNS in other studies was probably because of the short exposure times used.

Compared to *ADGRL1* mRNA, *ADGRL2* mRNA presented a substantial different organization because *ADGRL2* mRNA expression is ubiquitous and uniformly distributed in all tissues. The highest expression of *ADGRL2* was observed in placenta and lung, and the lowest were observed in brain and liver (Sugita et al., 1998). According to Ichtchenko et al. (1999), employing northern blot on adult human tissues, *ADGRL2* mRNA expression is detected almost in all tissues tested. The highest expression was detected in placenta, heart, lung, kidney, pancreas, spleen, and ovary. Moderate expression was seen in brain, liver, and testis. Weak expression was observed in the skeletal muscle and thymus and peripheral blood leukocytes did not presented *ADGRL2* mRNA expression.

According to Sugita et al. (1998), *ADGRL3* mRNA expression was only observed in the human brain. Subsequently, Ichtchenko et al. (1999) described that *ADGRL3* mRNA was expressed mainly in the brain. Weak expression was reported in heart, placenta, pancreas, kidney, and testis. Northern blot analysis of different human brain tissue samples performed by Arcos-Burgos et al. (2010) showed significant expression of *ADGRL3* mRNA in amygdala, caudate nucleus, cerebellum and cerebral cortex. Lower expression was found in corpus callosum, hippocampus, whole brain extract, occipital pole, frontal lobe, temporal lobe, and putamen. No expression was detected in thalamus, medulla and spinal cord. *In situ* hybridization in human brain of different ages revealed strong cytoplasmatic signals in the amygdala, caudate nucleus, pontine nucleus and in Purkinje cells of the cerebellum at all ages tested. Weak signals were detected in cingulate gyrus neurons in the 2- and 5-year old, but not in the 8- and 30-year old, and in *indusium griseum* neurons in the 2-year old. Areas of the brain that were labeled by *in situ* hybridization also were labeled by immunohistochemistry.

## Conclusion

The teneurin-latrophilin system is a remarkable model for the study of protein-receptor interactions. The study of this system gives us a window into the fascinating exchanges that occurred between prokaryotes and basal eukaryotes, the repurposing of bioactive molecules once they are acquired, and the increasing complexity of neuropeptidergic systems as metazoans evolved. In particular, the teneurin system is remarkable in the sense that it is possible to build a phylogenetic tree based on the distribution and temporal dynamics of *Ten* expression that will result in a similar tree based on sequence similarity. *Ten1* and *Ten4* genes, in the available models, share the early and widespread expression of the *C. elegans* teneurin that results in the long isoform. On the other hand, *Ten2* and *Ten3* are expressed late during development and became more pronounced in the nervous system and, in particular, in axonal guidance, resembling the *C. elegans* teneurin that results in the short isoform. The appearance of alternative regulation of teneurin expression after the divergence of Cnidaria/Ctenophora may have fundamentally impacted the way the teneurins acquired new functions and new corresponding distributions as nervous system complexity increased. In a similar vein, the appearance of isoforms before the nematode lineage likely facilitated the *Insecta* lineage to develop two teneurin paralogs that resemble the Ten1/Ten4 and the Ten2/Ten3 in terms of morphology, despite no direct homology between these proteins. This is a case of convergent evolution facilitated by a molecular event common to the species involved. It is interesting to question, however, how much causation there is between the increase in teneurin complexity in metazoans and the increase in complexity in sensory systems and in the nervous system as a whole. If certainly not the only promoter in that increasing complexity, it is hard to imagine that more complex sensory systems could have evolved without a system in place to ensure the correct connectivity, and the teneurins-latrophilins must have contributed in that process. If this is the case, teneurin-latrophilin interactions can be predicted to play an essential role in human physiology. As reviewed in this work, however, the study of those interactions is undermined by insufficient morphological data and a limited number of animal models employed. New studies are urged to fill the gaps and facilitate our understanding of this system.

## Author Contributions

LS wrote the text and reviewed the literature. GD designed and wrote the text and reviewed the literature. JH-J reviewed the literature. CC reviewed the literature. JB designed and wrote the text.

## Conflict of Interest Statement

The authors declare that the research was conducted in the absence of any commercial or financial relationships that could be construed as a potential conflict of interest.

## Footnotes

1 www.flyatlas.org

## References

[B1] Anderson G. R. Maxeiner S. Sando R. Tsetsenis T. Malenka R. C. Südhof T. C. (2017). Postsynaptic adhesion GPCR latrophilin-2 mediates target recognition in entorhinal-hippocampal synapse assembly. *J. Cell Biol.* 216 3831–3846. 10.1083/jcb.201703042 28972101 PMC5674891

[B2] Antinucci P. Nikolaou N. Meyer M. P. Hindges R. (2013). Teneurin-3 specifies morphological and functional connectivity of retinal ganglion cells in the vertebrate visual system. *Cell Rep.* 5 582–592. 10.1016/j.celrep.2013.09.045 24183672 PMC3898612

[B3] Arcos-Burgos M. Jain M. Acosta M. Shively S. Stanescu H. Wallis D. (2010). A common variant of the latrophilin 3 gene, LPHN3, confers susceptibility to ADHD and predicts effectiveness of stimulant medication. *Mol. Psychiatry* 15:1053. 10.1038/mp.2010.6 20157310

[B4] Baumgartner S. Chiquet-Ehrismann R. (1993). Tena, a Drosophila gene related to tenascin, shows selective transcript localization. *Mech. Dev.* 40 165–176. 7684246 10.1016/0925-4773(93)90074-8

[B5] Baumgartner S. Martin D. Hagios C. Chiquet-Ehrismann R. (1994). Tenm, a *Drosophila* gene related to tenascin, is a new pair-rule gene. *EMBO J.* 13 3728–3740. 8070401 10.1002/j.1460-2075.1994.tb06682.x PMC395283

[B6] Ben-Zur T. Feige E. Motro B. Wides R. (2000). The mammalian Odz gene family: homologs of a *Drosophila* pair-rule gene with expression implying distinct yet overlapping developmental roles. *Dev. Biol.* 217 107–120. 10625539 10.1006/dbio.1999.9532

[B7] Ben-Zur T. Wides R. (1999). Mapping Homologs of *Drosophila* odd Oz (odz): Doc4/Odz4to mouse chromosome 7, Odz1to mouse chromosome 11; and ODZ3 to human chromosome Xq25. *Genomics* 1 102–103. 10331952 10.1006/geno.1999.5798

[B8] Berns D. S. DeNardo L. A. Pederick D. T. Luo L. (2018). Teneurin-3 controls topographic circuit assembly in the hippocampus. *Nature* 554:328. 10.1038/nature25463 29414938 PMC7282895

[B9] Boucard A. A. Maxeiner S. Südhof T. C. (2014). Latrophilins function as heterophilic cell-adhesion molecules by binding to teneurins REGULATION BY ALTERNATIVE SPLICING. *J. Biol. Chem.* 289 387–402. 10.1074/jbc.M113.504779 24273166 PMC3879561

[B10] Carr O. P. Glendining K. A. Leamey C. A. Marotte L. R. (2013). Overexpression of Ten-m3 in the retina alters ipsilateral retinocollicular projections in the wallaby (*Macropus eugenii*). *Int. J. Dev. Neurosci.* 31 496–504. 10.1016/j.ijdevneu.2013.05.011 23747822

[B11] Carr O. P. Glendining K. A. Leamey C. A. Marotte L. R. (2014). Retinal overexpression of Ten-m3 alters ipsilateral retinogeniculate projections in the wallaby (*Macropus eugenii*). *Neurosci. Lett.* 566 167–171. 10.1016/j.neulet.2014.02.048 24602979

[B12] Chand D. Casatti C. A. de Lannoy L. Song L. Kollara A. Barsyte-Lovejoy D. (2013). C-terminal processing of the teneurin proteins: independent actions of a teneurin C-terminal associated peptide in hippocampal cells. *Mol. Cell. Neurosci.* 52 38–50. 10.1016/j.mcn.2012.09.006 23026563

[B13] Chand D. Colacci M. Dixon K. Kollara A. Brown T. J. Lovejoy D. A. (2014). C-terminal region of teneurin-1 co-localizes with the dystroglycan complex in adult mouse testes and regulates testicular size and testosterone production. *Histochem. Cell Biol.* 141 191–211. 10.1007/s00418-013-1154-1 24154551

[B14] Chen Y. Xu M. De Almeida R. Lovejoy D. A. (2013). Teneurin C-terminal associated peptides (TCAP): modulators of corticotropin-releasing factor (CRF) physiology and behavior. *Front. Neurosci.* 7:166. 10.3389/fnins.2013.00166 24062636 PMC3775549

[B15] Colacci M. De Almeida R. Chand D. Lovejoy S. R. Sephton D. Vercaemer B. (2015). Characterization of the teneurin C-terminal associated peptide (TCAP) in the vase tunicate, *Ciona intestinalis*: a novel peptide system associated with energy metabolism and reproduction. *Gen. Comp. Endocrinol.* 216 161–170. 10.1016/j.ygcen.2015.01.021 25687741

[B16] D’Aquila A. L. Hsieh A. H.-R. Hsieh A. H.-M. Almeida R. Lovejoy S. R. Lovejoy D. A. (2017). Expression and actions of corticotropin-releasing factor/diuretic hormone-like peptide (CDLP) and teneurin C-terminal associated peptide (TCAP) in the vase tunicate, *Ciona intestinalis*: antagonism of the feeding response. *Gen. Comp. Endocrinol.* 246 105–115. 10.1016/j.ygcen.2016.06.015 27292788

[B17] Davletov B. A. Shamotienko O. G. Lelianova V. G. Grishin E. V. Ushkaryov Y. A. (1996). Isolation and biochemical characterization of a Ca2+-independent α-latrotoxin-binding protein. *J. Biol. Chem.* 271 23239–23245. 8798521 10.1074/jbc.271.38.23239

[B18] Dehal P. Boore J. L. (2005). Two rounds of whole genome duplication in the ancestral vertebrate. *PLoS Biol.* 3:e314. 10.1371/journal.pbio.0030314 16128622 PMC1197285

[B19] Dharmaratne N. Glendining K. A. Young T. R. Tran H. Sawatari A. Leamey C. A. (2012). Ten-m3 is required for the development of topography in the ipsilateral retinocollicular pathway. *PLoS One* 7:e43083. 10.1371/journal.pone.0043083 23028443 PMC3446960

[B20] Doyle S. E. Scholz M. J. Greer K. A. Hubbard A. D. Darnell D. K. Antin P. B. (2006). Latrophilin-2 is a novel component of the epithelial-mesenchymal transition within the atrioventricular canal of the embryonic chicken heart. *Dev. Dyn.* 235 3213–3221. 17016846 10.1002/dvdy.20973

[B21] Drabikowski K. Trzebiatowska A. Chiquet-Ehrismann R. (2005). ten-1, an essential gene for germ cell development, epidermal morphogenesis, gonad migration, and neuronal pathfinding in *Caenorhabditis elegans*. *Dev. Biol.* 282 27–38. 15936327 10.1016/j.ydbio.2005.02.017

[B22] Fascetti N. Baumgartner S. (2002). Expression of *Drosophila* Ten-a, a dimeric receptor during embryonic development. *Mech. Dev.* 114 197–200. 12175511 10.1016/s0925-4773(02)00055-2

[B23] Graumann R. Di Capua G. A. Oyarzún J. E. Vásquez M. A. Liao C. Brañes J. A. (2017). Expression of teneurins is associated with tumor differentiation and patient survival in ovarian cancer. *PLoS One* 12:e0177244. 10.1371/journal.pone.0177244 28472127 PMC5417686

[B24] Hamann J. Aust G. Araç D. Engel F. B. Formstone C. Fredriksson R. (2015). International union of basic and clinical pharmacology. XCIV. adhesion G protein–coupled receptors. *Pharmacol. Rev.* 67 338–367. 10.1124/pr.114.009647 25713288 PMC4394687

[B25] Hong W. Mosca T. J. Luo L. (2012). Teneurins instruct synaptic partner matching in an olfactory map. *Nature* 484:201. 10.1038/nature10926 22425994 PMC3345284

[B26] Husić M. Barsyte-Lovejoy D. Lovejoy D. A. (2019). Teneurin C-terminal associated peptide (TCAP)-1 and latrophilin interaction in HEK293 cells: evidence for modulation of intercellular adhesion. *Front. Endocrinol.* 10:22. 10.3389/fendo.2019.00022 30774623 PMC6367273

[B27] Ichtchenko K. Bittner M. A. Krasnoperov V. Little A. R. Chepurny O. Holz R. W. (1999). A novel ubiquitously expressed α-latrotoxin receptor is a member of the CIRL family of G-protein-coupled receptors. *J. Biol. Chem.* 274 5491–5498. 10026162 10.1074/jbc.274.9.5491

[B28] Ichtchenko K. Khvotchev M. Kiyatkin N. Simpson L. Sugita S. Sudhof T. C. (1998). alpha-latrotoxin action probed with recombinant toxin: receptors recruit alpha-latrotoxin but do not transduce an exocytotic signal. *EMBO J.* 17 6188–6199. 10.1093/emboj/17.21.6188 9799228 PMC1170945

[B29] Jackson V. A. del Toro D. Carrasquero M. Roversi P. Harlos K. Klein R. (2015). Structural basis of latrophilin-FLRT interaction. *Structure* 23 774–781. 10.1016/j.str.2015.01.013 25728924 PMC4396693

[B30] Kenzelmann D. Chiquet-Ehrismann R. Leachman N. T. Tucker R. P. (2008). Teneurin-1 is expressed in interconnected regions of the developing brain and is processed in vivo. *BMC Dev. Biol.* 8:30. 10.1186/1471-213X-8-30 18366734 PMC2289808

[B31] King N. Westbrook M. J. Young S. L. Kuo A. Abedin M. Chapman J. (2008). The genome of the choanoflagellate *Monosiga brevicollis* and the origin of metazoans. *Nature* 451:783. 10.1038/nature06617 18273011 PMC2562698

[B32] Krasnoperov V. G. Beavis R. Chepurny O. G. Little A. R. Plotnikov A. N. Petrenko A. G. (1996). The calcium-independent receptor of α-latrotoxin is not a neurexin. *Biochem. Biophys. Res. Commun.* 227 868–875. 8886023 10.1006/bbrc.1996.1598

[B33] Krasnoperov V. G. Bittner M. A. Beavis R. Kuang Y. Salnikow K. V. Chepurny O. G. (1997). α-Latrotoxin stimulates exocytosis by the interaction with a neuronal G-protein-coupled receptor. *Neuron* 18 925–937. 9208860 10.1016/s0896-6273(00)80332-3

[B34] Langenhan T. Prömel S. Mestek L. Esmaeili B. Waller-Evans H. Hennig C. (2009). Latrophilin signaling links anterior-posterior tissue polarity and oriented cell divisions in the *C. elegans* embryo. *Dev. Cell* 17 494–504. 10.1016/j.devcel.2009.08.008 19853563 PMC2819344

[B35] Leamey C. A. Glendining K. A. Kreiman G. Kang N.-D. Wang K. H. Fassler R. (2007a). Differential gene expression between sensory neocortical areas: potential roles for Ten_m3 and Bcl6 in patterning visual and somatosensory pathways. *Cereb. Cortex* 18 53–66. 17478416 10.1093/cercor/bhm031

[B36] Leamey C. A. Merlin S. Lattouf P. Sawatari A. Zhou X. Demel N. (2007b). Ten_m3 regulates eye-specific patterning in the mammalian visual pathway and is required for binocular vision. *PLoS Biol.* 5:e241. 17803360 10.1371/journal.pbio.0050241 PMC1964777

[B37] Lelianova V. G. Davletov B. A. Sterling A. Rahman M. A. Grishin E. V. Totty N. F. (1997). α-Latrotoxin receptor, latrophilin, is a novel member of the secretin family of G protein-coupled receptors. *J. Biol. Chem.* 272 21504–21508. 9261169 10.1074/jbc.272.34.21504

[B38] Leon K. Lu Y. C. Nazarko O. Sando R. Salzman G. Südhof T. (2017). Structural and functional studies of latrophilin-family adhesion G-protein coupled receptors. *FASEB J.* 31 667–674.

[B39] Levine A. Bashan-Ahrend A. Budai-Hadrian O. Gartenberg D. Menasherow S. Wides R. (1994). Odd Oz: a novel *Drosophila* pair rule gene. *Cell* 77 587–598. 7514504 10.1016/0092-8674(94)90220-8

[B40] Levine A. Weiss C. Wides R. (1997). Expression of the pair-rule gene odd Oz (odz) in imaginal tissues. *Dev. Dyn.* 209 1–14. 9142491 10.1002/(SICI)1097-0177(199705)209:1<1::AID-AJA1>3.0.CO;2-M

[B41] Li H. Bishop K. M. O’leary D. D. (2006). Potential target genes of EMX2 include Odz/Ten-M and other gene families with implications for cortical patterning. *Mol. Cell. Neurosci.* 33 136–149. 16919471 10.1016/j.mcn.2006.06.012

[B42] Li J. Shalev-Benami M. Sando R. Jiang X. Kibrom A. Wang J. (2018). Structural basis for teneurin function in circuit-wiring: a toxin motif at the synapse. *Cell* 173 735.e15–748.e15. 10.1016/j.cell.2018.03.036 29677516 PMC5912346

[B43] Lossie A. C. Nakamura H. Thomas S. E. Justice M. J. (2005). Mutation of l7Rn3 shows that Odz4 is required for mouse gastrulation. *Genetics* 169 285–299. 15489520 10.1534/genetics.104.034967 PMC1448887

[B44] Lovejoy D. A. Al Chawaf A. Cadinouche M. A. (2006). Teneurin C-terminal associated peptides: an enigmatic family of neuropeptides with structural similarity to the corticotropin-releasing factor and calcitonin families of peptides. *Gen. Comp. Endocrinol.* 148 299–305. 16524574 10.1016/j.ygcen.2006.01.012

[B45] Lu Y. C. Nazarko O. V. Sando R. III Salzman G. S. Li N.-S. Südhof T. C. (2015). Structural basis of latrophilin-FLRT-UNC5 interaction in cell adhesion. *Structure* 23 1678–1691. 10.1016/j.str.2015.06.024 26235030 PMC4851429

[B46] Matsushita H. Lelianova V. G. Ushkaryov Y. A. (1999). The latrophilin family: multiply spliced G protein-coupled receptors with differential tissue distribution. *FEBS Lett.* 443 348–352. 10025961 10.1016/s0014-5793(99)00005-8

[B47] Mee C. J. Tomlinson S. R. Perestenko P. V. Pomerai D. Duce I. R. Usherwood P. N. R. (2004). Latrophilin is required for toxicity of black widow spider venom in *Caenorhabditis elegans*. *Biochem. J.* 378 185–191. 14594448 10.1042/BJ20031213 PMC1223931

[B48] Meyer A. Van de Peer Y. (2005). From 2R to 3R: evidence for a fish-specific genome duplication (FSGD). *Bioessays* 27 937–945. 10.1002/bies.20293 16108068

[B49] Mieda M. Kikuchi Y. Hirate Y. Aoki M. Okamoto H. (1999). Compartmentalized expression of zebrafish ten-m3 and ten-m4, homologues of the *Drosophila* tenm/odd Oz gene, in the central nervous system. *Mech. Dev.* 87 223–227. 10495292 10.1016/s0925-4773(99)00155-0

[B50] Minet A. D. Chiquet-Ehrismann R. (2000). Phylogenetic analysis of teneurin genes and comparison to the rearrangement hot spot elements of *E. coli*. *Gene* 257 87–97. 11054571 10.1016/s0378-1119(00)00388-7

[B51] Minet A. D. Rubin B. P. Tucker R. P. Baumgartner S. Chiquet-Ehrismann R. (1999). Teneurin-1, a vertebrate homologue of the *Drosophila* pair-rule gene ten-m, is a neuronal protein with a novel type of heparin-binding domain. *J. Cell Sci.* 112(Pt 12), 2019–2032. 10341219 10.1242/jcs.112.12.2019

[B52] Mörck C. Vivekanand V. Jafari G. Pilon M. (2010). *C. elegans* ten-1 is synthetic lethal with mutations in cytoskeleton regulators, and enhances many axon guidance defective mutants. *BMC Dev. Biol.* 10:55. 10.1186/1471-213X-10-55 20497576 PMC2887410

[B53] Ohno S. (2013). *Evolution By Gene Duplication.* Berlin: Springer Science & Business Media.

[B54] Oohashi T. Zhou X.-H. Feng K. Richter B. Mörgelin M. Perez M. T. (1999). Mouse ten-m/Odz is a new family of dimeric type II transmembrane proteins expressed in many tissues. *J. Cell Biol.* 145 563–577. 10225957 10.1083/jcb.145.3.563 PMC2185078

[B55] Otaki J. M. Firestein S. (1999). Neurestin: putative transmembrane molecule implicated in neuronal development. *Dev. Biol.* 212 165–181. 10419693 10.1006/dbio.1999.9310

[B56] Qian X. Barsyte-Lovejoy D. Wang L. Chewpoy B. Gautam N. Al Chawaf A. (2004). Cloning and characterization of teneurin C-terminus associated peptide (TCAP)-3 from the hypothalamus of an adult rainbow trout (*Oncorhynchus mykiss*). *Gen. Comp. Endocrinol.* 137 205–216. 15158132 10.1016/j.ygcen.2004.02.007

[B57] Rubin B. P. Tucker R. P. Brown-Luedi M. Martin D. Chiquet-Ehrismann R. (2002). Teneurin 2 is expressed by the neurons of the thalamofugal visual system in situ and promotes homophilic cell-cell adhesion in vitro. *Development* 129 4697–4705. 12361962 10.1242/dev.129.20.4697

[B58] Rubin B. P. Tucker R. P. Martin D. Chiquet-Ehrismann R. (1999). Teneurins: a novel family of neuronal cell surface proteins in vertebrates, homologous to the *Drosophila* pair-rule gene product Ten-m. *Dev. Biol.* 216 195–209. 10.1006/dbio.1999.9503 10588872

[B59] Sacerdot C. Louis A. Bon C. Berthelot C. Crollius H. R. (2018). Chromosome evolution at the origin of the ancestral vertebrate genome. *Genome Biol.* 19:166. 10.1186/s13059-018-1559-1 30333059 PMC6193309

[B60] Scholz N. Gehring J. Guan C. Ljaschenko D. Fischer R. Lakshmanan V. (2015). The adhesion GPCR latrophilin/CIRL shapes mechanosensation. *Cell Rep.* 11 866–874. 10.1016/j.celrep.2015.04.008 25937282

[B61] Silva J.-P. Lelianova V. G. Ermolyuk Y. S. Vysokov N. Hitchen P. G. Berninghausen O. (2011). Latrophilin 1 and its endogenous ligand Lasso/teneurin-2 form a high-affinity transsynaptic receptor pair with signaling capabilities. *Proc. Natl. Acad. Sci. U.S.A.* 108 12113–12118. 10.1073/pnas.1019434108 21724987 PMC3141932

[B62] Sugita S. Ichtchenko K. Khvotchev M. Südhof T. C. (1998). α-Latrotoxin receptor CIRL/latrophilin 1 (CL1) defines an unusual family of ubiquitous G-protein-linked receptors G-PROTEIN COUPLING NOT REQUIRED FOR TRIGGERING EXOCYTOSIS. *J. Biol. Chem.* 273 32715–32724. 9830014 10.1074/jbc.273.49.32715

[B63] Tews D. Fromme T. Keuper M. Hofmann S. Debatin K.-M. Klingenspor M. (2017). Teneurin-2 (TENM2) deficiency induces UCP1 expression in differentiating human fat cells. *Mol. Cell. Endocrinol.* 443 106–113. 10.1016/j.mce.2017.01.015 28088466

[B64] Tews D. Schwar V. Scheithauer M. Weber T. Fromme T. Klingenspor M. (2014). Comparative gene array analysis of progenitor cells from human paired deep neck and subcutaneous adipose tissue. *Mol. Cell. Endocrinol.* 395 41–50. 10.1016/j.mce.2014.07.011 25102227

[B65] Torres-da-Silva K. Tessarin G. Dias C. Guiati I. Ervolino E. Gonçalves A. (2017). Teneurin-2 presence in rat and human odontoblasts. *PLoS One* 12:e0184794. 10.1371/journal.pone.0184794 28926618 PMC5604987

[B66] Tran H. Sawatari A. Leamey C. A. (2015). The glycoprotein Ten-m3 mediates topography and patterning of thalamostriatal projections from the parafascicular nucleus in mice. *Eur. J. Neurosci.* 41 55–68. 10.1111/ejn.12767 25406022

[B67] Trzebiatowska A. Topf U. Sauder U. Drabikowski K. Chiquet-Ehrismann R. (2008). *Caenorhabditis elegans* teneurin, ten-1, is required for gonadal and pharyngeal basement membrane integrity and acts redundantly with integrin ina-1 and dystroglycan dgn-1. *Mol. Biol. Cell* 19 3898–3908. 10.1091/mbc.E08-01-0028 18632986 PMC2526705

[B68] Tucker R. P. (2013). Horizontal gene transfer in choanoflagellates. *J. Exp. Zool. Part B* 320 1–9. 10.1002/jez.b.22480 22997182

[B69] Tucker R. P. Beckmann J. Leachman N. T. Schöler J. Chiquet-Ehrismann R. (2012). Phylogenetic analysis of the teneurins: conserved features and premetazoan ancestry. *Mol. Biol. Evol.* 29 1019–1029. 10.1093/molbev/msr271 22045996 PMC3278476

[B70] Tucker R. P. Chiquet-Ehrismann R. (2006). Teneurins: a conserved family of transmembrane proteins involved in intercellular signaling during development. *Dev. Biol.* 290 237–245. 16406038 10.1016/j.ydbio.2005.11.038

[B71] Tucker R. P. Chiquet-Ehrismann R. Chevron M. P. Martin D. Hall R. J. Rubin B. P. (2001). Teneurin-2 is expressed in tissues that regulate limb and somite pattern formation and is induced in vitro and in situ by FGF8. *Dev. Dyn.* 220 27–39. 11146505 10.1002/1097-0177(2000)9999:9999<::AID-DVDY1084>3.0.CO;2-B

[B72] Tucker R. P. Kenzelmann D. Trzebiatowska A. Chiquet-Ehrismann R. (2007). Teneurins: transmembrane proteins with fundamental roles in development. *Int. J. Biochem. Cell Biol.* 39 292–297. 17095284 10.1016/j.biocel.2006.09.012

[B73] Tucker R. P. Martin D. Kos R. Chiquet-Ehrismann R. (2000). The expression of teneurin-4 in the avian embryo. *Mech. Dev.* 98 187–191. 11044628 10.1016/s0925-4773(00)00444-5

[B74] van der Voet M. Harich B. Franke B. Schenck A. (2016). ADHD-associated dopamine transporter, latrophilin and neurofibromin share a dopamine-related locomotor signature in *Drosophila*. *Mol. Psychiatry* 21:565. 10.1038/mp.2015.55 25962619 PMC4804182

[B75] Wang L. Rotzinger S. Al Chawaf A. Elias C. F. Baršytë-Lovejoy D. Qian X. (2005). Teneurin proteins possess a carboxy terminal sequence with neuromodulatory activity. *Mol. Brain Res.* 133 253–265. 10.1016/j.molbrainres.2004.10.019 15710242

[B76] Wang X. Z. Kuroda M. Sok J. Batchvarova N. Kimmel R. Chung P. (1998). Identification of novel stress-induced genes downstream of chop. *EMBO J.* 17 3619–3630. 9649432 10.1093/emboj/17.13.3619 PMC1170698

[B77] Willson J. Amliwala K. Davis A. Cook A. Cuttle M. F. Kriek N. (2004). Latrotoxin receptor signaling engages the UNC-13-dependent vesicle-priming pathway in *C. elegans*. *Curr. Biol.* 14 1374–1379. 15296755 10.1016/j.cub.2004.07.056

[B78] Wilson R. Ainscough R. Anderson K. Baynes C. Berks M. Bonfield J. (1994). 2.2 Mb of contiguous nucleotide sequence from chromosome III of *C. elegans*. *Nature* 368 32–38. 10.1038/368032a0 7906398

[B79] Woelfle R. D’Aquila A. L. Pavloviæ T. Husiæ M. Lovejoy D. A. (2015). Ancient interaction between the teneurin C-terminal associated peptides (TCAP) and latrophilin ligand-receptor coupling: a role in behavior. *Front. Neurosci.* 9:146. 10.3389/fnins.2015.00146 25964737 PMC4408839

[B80] Young T. R. Bourke M. Zhou X. Oohashi T. Sawatari A. Fässler R. (2013). Ten-m2 is required for the generation of binocular visual circuits. *J. Neurosci.* 33 12490–12509. 10.1523/JNEUROSCI.4708-12.2013 23884953 PMC6618674

[B81] Young T. R. Leamey C. A. (2009). Teneurins: important regulators of neural circuitry. *Int. J. Biochem. Cell Biol.* 41 990–993. 10.1016/j.biocel.2008.06.014 18723111

[B82] Zhang D. de Souza R. F. Anantharaman V. Iyer L. M. Aravind L. (2012). Polymorphic toxin systems: comprehensive characterization of trafficking modes, processing, mechanisms of action, immunity and ecology using comparative genomics. *Biol. Dir.* 7:18. 10.1186/1745-6150-7-18 22731697 PMC3482391

[B83] Zheng L. Michelson Y. Freger V. Avraham Z. Venken K. J. Bellen H. J. (2011). *Drosophila* ten-m and filamin affect motor neuron growth cone guidance. *PLoS One* 6:e22956. 10.1371/journal.pone.0022956 21857973 PMC3152545

[B84] Zhou X.-H. Brandau O. Feng K. Oohashi T. Ninomiya Y. Rauch U. (2003). The murine Ten-m/Odz genes show distinct but overlapping expression patterns during development and in adult brain. *Gene Exp. Patterns* 3 397–405. 12915301 10.1016/s1567-133x(03)00087-5

[B85] Zuko A. Oguro-Ando A. Post H. Taggenbrock R. L. Van Dijk R. E. Altelaar A. (2016). Association of cell adhesion molecules contactin-6 and latrophilin-1 regulates neuronal apoptosis. *Front. Mol. Neurosci.* 9:143 10.3389/fnmol.2016.00143 PMC5156884 28018171

