# Supplementary material for: Nomenclature and Comparative Morphology of the Teneurin/TCAP/ADGRL Protein Families
Source: Front Neurosci. 2019 May 3;13:425. doi: 10.3389/fnins.2019.00425 (PMC6510184; doi:10.3389/fnins.2019.00425)
Supplement: Supplementary file 1 [file Table_1.DOCX]

**Supplementary material - Table 01**

| **Target** | **Antibody name** | **Species raised** | **Company** | **Reference** | **Information extracted from the cited article** |
| --- | --- | --- | --- | --- | --- |
| ***C. elegans*** | | | | | |
| *C. elegans* TEN-S/TEN-L | anti-ten1 (C-terminal) / anti-ten1 (N-terminal) | rabbit polyclonal | Neosystem | Drabikowski et al, 2005 | "Anti-Ten-1 antibodies were raised against the Ten-1-specific peptides from the N-terminus of the long variant (MFQHRTTNAQGPPPNRPMPR) and the common C-terminus (PAHQSGLLASVHSWKFRKSE). The peptides were synthesized and the rabbits immunized at Neosystem.” |
| ***D. melanogaster*** | | | | | |
| *D. melanogaster* TEN-m | anti-MAb20 | mouse monoclonal | non-commercial | Levine et al, 1994; Levine, 1997; Zheng et al, 2011; Hong et al, 2012 | "Monoclonal antibodies against a collection of Drosophila proteins immunopurified (...) A number of antibodies against specific proteins, including MAb20, were generated." |
| *D. melanogaster* TEN-m | anti-mab113 | mouse monoclonal | non-commercial | Baumgartner et al, 1994 | "Fusion proteins c13.2 and c5.4 correspond to aminoacids 56-251 and 912-1391 respectively of the tenm sequence.The fusion proteins were used to generate monoclonal antibodies and rabbit polyclonal antisera.(...) For embryo staining, mab113 was used." |
| *D. melanogaster* TEN-a | anti-Ten-a | rabbit polyclonal | non-commercial | Fascetti and Baumgartner, 2002 | "A 2.3-kbEco RI fragment from cDT17.4 was expressed in bacteria, the fusion protein was cut out from an agarose gel, and the eluted fusion protein was injected into rabbits." |

| ***C. intestinalis*** | | | | | |
| --- | --- | --- | --- | --- | --- |
| *C. intestinalis* Ten1 | TNR5 (Ciona TCAP N-terminal Region Rabbit 5) | rabbit polyclonal | non-commercial | D'Aquila et al, 2017 | "The N and C terminal fragments of the (...) TCAP peptide were used as haptens for the development of the antisera. Briefly, (...) N-terminal TCAP, C-terminal TCAP (...) haptens were injected into three rabbits each to immunize for antisera production." |
| ***G. gallus*** | | | | | |
| *G. gallus* TEN1 | anti-tenascin c (antiTn60) | mouse monoclonal | non-commercial | Pearson et al, 1988; Minet et al, 1999 | "The N-terminal part of chicken tenascin-C (nucleotides 1-794 of the tenascin-C EMBL database entry M23121) including its 5′ UTR, and the coding sequence for the signal and propeptide as well as the 153 N-terminal amino acids carrying the epitope for the monoclonal antibody anti-TN6" |
| *G. gallus* TEN1 | anti-ICD teneurin 1 | rabbit polyclonal | non-commercial | Kenzelmann et al., 2008 | "...the 160 N-terminal amino acids of the ICD of chicken teneurin-1" |
| *G. gallus* TEN1 | anti-ECD teneurin 1 | rabbit polyclonal | non-commercial | Kenzelmann et al., 2008 | "...the 300 C-terminal amino acids of the extracellular domain of chicken teneurin-1" |
| *G. gallus* TEN1 | anti-EGF teneurin 1 | rabbit polyclonal | non-commercial | Kenzelmann et al., 2008 | "...a fusion of the N-terminus of tenascin-C (for protein purification purposes) and the 8 EGF-like domains of chicken teneurin-1" |

| *G. gallus* TEN2 | anti-chicken teneurin 2 | rabbit polyclonal | non-commercial | Rubin et al, 1999; Tucker et al, 20001; Rubin et al, 2002; Kenzelmann et al, 2008 | "anti-Tenascin-C/Teneurin-2 Fusion Protein (TN/ten-2) (..) The N-terminal part of chicken tenascin-C (nucleotides 1–794 tenascin-C EMBL database entry M23121), which contains the epitope for the monoclonal antibody anti-TN60 (Pearson et al. , 1988), was fused to the extracellular half of chicken teneurin-2 (amino acids 429 – 831). (...) The affinity-purified protein TN/ten-2 was injected into rabbits for antibody production." |
| --- | --- | --- | --- | --- | --- |
| ***M. musculus*** | | | | | |
| *M. musculus* TEN1 | anti-mouse Ten-m1 | rabbit polyclonal | non-commercial | Oohashi et al, 1999; Zhou et al, 2003 | "...extracellular domain of Ten-m1" |
| *M. musculus* TEN1 | anti-teneurin-1 | mouse monoclonal | ABNOVA | Chand et al, 2013; Chand et al, 2014 |  |
| *M. musculus* TEN2 | anti-mouse Ten-m/Odz2 | rabbit polyclonal | non-commercial | Zhou et al, 2003; Young et al, 2013 | "... against extracellular parts of Ten-m/Odz2" |
| *M. musculus* TEN3 | anti-mouse Ten-m/Odz3 | rabbit polyclonal | non-commercial | Zhou et al, 2003; Leamey et al, 2015 | "... against extracellular parts of (...) Ten-m/Odz3" |
| *M. musculus* TEN3 | anti-Ten3IC | rabbit polyclonal | non-commercial | Berns et al, 2018 | "... custom antibody against aminoacids 163-173" |
| *M. musculus* TEN3 | anti-Ten3EC | rabbit polyclonal | non-commercial | Berns et al, 2018 | "... custom antibody against aminoacids 346-364" |
| *M. musculus* TEN4 | anti-mouse Ten-m/Odz4 | rabbit polyclonal | non-commercial | Zhou et al, 2003 | "... against extracellular parts of (...) Ten-m/Odz4" |
| *M. musculus* TEN4 | anti-DOC4 | rabbit polyclonal | non-commercial | Wang et al, 1998 | "peptide encoded by na Nco I fragment (nucleotides 7791–8237) from the 3' end of the cDNA" |
| *M. musculus* TEN4 | anti-Ten-4 (DOC4-M17): sc-10970 | goat polyclonal | Santa Cruz Biotechnology | Graumman et al, 2017 |  |
| *M. musculus* TCAP-1 | anti- N-terminal TCAP1 (TNR308) | rabbit polyclonal | non-commercial | Chand et al, 2013; Chand et al, 2014; Colacci et al, 2015 | "The sequences used as haptens were KLH-pEQLLGTGRVQGYDGYFVLSVEQYLE-OH (pE: pyroglutamic acid) and KLH-VLSVEQYLELSDSANNIHFMRQSEI-NH2. (...) Two un-conjugated peptide sequences of N-TCAP-1 and C-TCAP-1 were used for antisera specificity studies." |
| *M. musculus* TCAP-1 | anti- C-terminal TCAP1 (TCR108) | rabbit polyclonal | non-commercial | Chand et al, 2013; Colacci et al, 2015 |  |
| *M. musculus* TCAP-1 | anti-TCAP-1 | mouse monoclonal | ProMab Biotechnologies | Chand et al, 2014 |  |
| ***H. sapiens*** | | | | | |
| *H. sapiens* TEN2 | anti- teneurin-2 (N-13): sc-165674 | goat polyclonal | Santa Cruz Biotechnology, | Torres-da-Silva et al, 2017 |  |
| *H. sapiens* TEN2 | anti-Ten-2 (HPA038420) | rabbit polyclonal | Sigma-Aldrich | Graumman et al, 2017 |  |
| *H. sapiens* TEN4 | anti-Ten-4 (DOC4-T15): sc-103484 | goat polyclonal | Santa Cruz Biotechnology, | Graumman et al, 2017 |  |

Supplementary material - Table 1: List of antibodies used in the reviewed articles. The table presents a comprehensive list of antibodies used in immunohistochemistry experiments cited in this review. The original articles provided the primary source of information enclosed in the table, but additional aspects of commercial antibodies were obtained in their respective datasheets available online.
