# Supplementary material for: Nomenclature and Comparative Morphology of the Teneurin/TCAP/ADGRL Protein Families
Source: Front Neurosci. 2019 May 3;13:425. doi: 10.3389/fnins.2019.00425 (PMC6510184; doi:10.3389/fnins.2019.00425)
Supplement: Supplementary file 2 [file Table_2.DOCX]

**Supplementary material - Table 02**

| **Source** | **Reference** | **Information extracted from the cited article** |
| --- | --- | --- |
| ***D. melanogaster*** | | |
| *D. melanogaster* | Levine et al, 1994 | "Probes were derived from many portion of cDNA probes isolated" |
| *D. melanogaster Ten-a* | Baumgartner et al, 1993 | "To determine whether Drosophila contains a gene homologous to tenascin, the polymerase chain reaction (PCR) was performed using degenerated primers derived from the EGF portion of chicken tenascin" |
| *D. melanogaster Ten-a* | Baumgartner et al, 1994 | "...probe coding for the EGF-like domain of the Tena and whole Tenm" |
| *D. melanogaster Ten-m* | Baumgartner et al, 1994 | "...probe coding for the EGF-like domain of the Tena and whole Tenm" |
| ***C. intestinalis*** | | |
| *C.* intestinalis TCAP-1 | D'Aquila et al, 2017 | "TCAP primers were: forward; (5’-CGCCACCGTTTATCATCTT-3’) and reverse; (5’-CTTTAACAAAGCGCATGTTTC-3’)" |
| ***D. rerio*** | | |
| *D. rerio ten3* | Mieda et al, 1999 | "cDNA clones covering ORFs of ten-m3 and ten-m4 were obtained by screening a 18-20 hpf zebrafish embryo cDNA library" |
| *D. rerio ten3* | Antinucci et al, 2013 | "a 981 bp cDNA fragment (ORF 7034-8014) was cloned into a StrataClone Blunt PCR Cloning Vector pSC-B-amp/kan (Agilent Technologies), and the orientation of the insertion was determined by DNA sequencing. The sequences of primers used to amplify the fragment through PCR are as follows: forward primer 5'-GGGACTATGACATTCAAG CAGGTC-3'; reverse primer 5'-CATTGTTGGCACTGTCGGCCAG-3'" |
| *D. rerio ten4* | Mieda et al, 1999 | "cDNA clones covering ORFs of ten-m3 and ten-m4 were obtained by screening a 18-20 hpf zebrafish embryo cDNA library" |
| ***G. gallus*** | | |
| *G. gallus TEN1* | Rubin et al, 1999 | "...teneurin-1 RNA probe corresponded to a 1332-bp sequence encoding amino acids 177– 621" |
| *G. gallus TEN1* | Minet et al, 1999 | "In situ hybridization with [α-35 S]dCTP labeled teneurin-1 cDNA (encoding the region separating the EGF-like repeats and the YD-repeats from aa 868-1330) " |
| *G. gallus TEN1* | Kenzelmann et al, 2008 | "... 628 bp within the ICD (N-terminal intracellular domain)" |
| *G. gallus TEN2* | Rubin et al, 1999 | Northern Blot probe: "... 850 bp of the EGF-like repeat encoding sequence of teneurin 2" |
| *G. gallus TEN2* | Rubin et al, 1999; Tucker et al, 2001; Rubin et al, 2002 | ISH probe: "... teneurin-2 cDNA (encoding the EGF-like repeats from amino acid 695 to amino acid 823)" |
| *G. gallus TEN2* | Tucker et al, 2001 | TEN2L: "...corresponding to a 300-bp sequence after the EGF-like repeats in the long form of teneurin-2" |
| *G. gallus TEN4* | Tucker et al, 2000 | "The first primer set was M13 and 5’- AGACACAGCGCACCATACTGC 3’ and the second one T7 and 5’ -GCTGGATGTCCGTGTAACGCC-3’. This approach yielded cDNA clone pTen4b with a length of 604 bp. This clone was used to label a probe for in situ hybridization" |
| ***M. musculus*** | | |
| *M. musculus Ten1* | Oohashi et al, 1999 | "... a fragment ranging from nucleotide 75 to 1833" |
| *M. musculus Ten1* | Zhou et al, 2003 | "As templates subcloned cDNAs of Ten-m/Odz1 (sequence nucleotide (nt) 959-1200" |
| *M. musculus Ten1* | Li et al, 2006 | "... primer pair, (+) 5′-GAGTGGGATCCTGGAAAGATG-3′and (− ) 5′-TTCTTCCCCTTCCTGTAGCCT-3′" |
| *M. musculus Ten1* | Chand et al, 2013 | "... teneurin-1, forward 5′-gtgtcacctgatggcaccctctat-3′, reverse 5′-tcctgggtatgtcatcaaggccaa-3′ (402 bases)" |
| *M. musculus Ten2* | Oohashi et al, 1999 | "... a fragment ranging from nucleotide 1 to 2006" |
| *M. musculus Ten2* | Zhou et al, 2003 | "(...) Ten-m/Odz2 (sequence nucleotide (nt) 816-1420" |
| *M. musculus Ten2* | Li et al, 2006 | "Odz2 was obtained by subcloning the SalI–SacI I fragment of IMAGE Clone #4507440" |
| *M. musculus Ten2* | Young et al, 2013 | "Ten-m2 forward (fwd), 5′-CTCTATGACCCCCTCACCAA-3′; Ten-m2 reverse (rev), 5′-CCAGCTCTTCACGTCTGTCA-3′" |
| *M. musculus Ten3* | Oohashi et al, 1999 | "... fragment ranging from nucleotide 762 to 1408" |
| *M. musculus Ten3* | Ben-Zur et al, 2000 | "As an antisense probe, anEcoRI subclone ofOdz3 (clone917V) in KS1 (Stratagene), spanning nucleotides 3400 to 2300 bp from the 39end of theOdz3message, was linearized at thePst Ipolylinker site." |
| *M. musculus Ten3* | Zhou et al, 2003 | "(...) Ten-m/Odz3 (sequence nucleotide (nt) 853-1108" |
| *M. musculus Ten3* | Li et al, 2006 | "To make theOdz3 riboprobe, IMAGE Clone #917326 (ResGen) was used directly as it does not contain poly A." |
| *M. musculus Ten3* | Leamey et al, 2007; Dharmaratne et al, 2012; Tran et al, 2015; Leamey et al, 2015 | "... 200bp-long digoxigenin-labelled riboprobes to sense and antisense Ten-m3 sequences" |
| *M. musculus Ten3* | Berns et al, 2018 | "Ten3 probe containing base pairs 4124-4953 of Ten3 mRNA" |
| *M. musculus Ten4* | Wang et al, 1998 | "DOC4 primer 1, AGTACAAGATGGATG-AGGATGGCTC; primer 2, TGGCTGCTCTTGCTGGATACTC" |
| *M. musculus Ten4* | Oohashi et al, 1999 | "... a fragment ranging from nucleotide 1 to 1108" |
| *M. musculus Ten4* | Zhou et al, 2003 | "(...) Ten-m/Odz4 (sequence nucleotide (nt) 1096-1305" |
| *M. musculus Ten4* | Lossie et al, 2005 | Northern blot probe: "The 3.11 probe consists mostly of 5' UTR with some protein coding sequence, while the 6.2.5 probe is derived entirely from the coding region." |
| *M. musculus Ten4* | Lossie et al, 2005 | ISH probe: "We used a probe corresponding to nucleotides 148–946" |
| *M. musculus Ten4* | Li et al, 2006 | "Odz4 riboprobe was generated by subcloning the SalI–Kpn I fragment of IMAGE Clone #4022173 (ResGen) into pBluescript" |
| M. musculus *TCAP-1* | Chand et al, 2013 | "... mouse TCAP-1, forward 5′-ttcatttccttggatcagcttcctatg-3′, reverse 5′-aagctgctgcttttctccctctgtcca-3′ (585 bases)" |
| ***R. norvegicus*** | | |
| *R. norvegicus Ten2* | Otaki et al, 1999 | "... the 361-bp PCR product amplified using primers 59-CGTCCGCTCGAGAATGGATGTGAAGGATCGGCGACAT-39 and 59-ATAGTTTAGCGGCCGCGTGCTCAGGGAGTAGCCCTGGTGG-39" |
| *R. norvegicus Ten2* | Torres-da-Silva et al, 2017 | 495 bp: Forward primer 5'-tgtgactgcaaaaacgatgtcaac-3' and reverse primer 5'-tcccatcataagtcatgaggcccagc-3’ |
| *R. norvegicus TCAP-1* | Wang et al, 2005 | "... riboprobes were generated from 350-bp cDNA of mouse teneurin exon 31 (including TCP-1 portion)" |
| *R. norvegicus TCAP-2* | Torres-da-Silva et al, 2017 | 496bp: Forward primer 5'-gacaagatgcactacagcatcgag-3' and reverse primer 5'-ccatctcattctgtcttaagaactgg-3' |
| ***H. sapiens*** | | |
| *H. sapiens TEN2* | Torres-da-Silva et al, 2017 | 495bp: Forward primer 5'-gagaacaatgtcatccttcgaatc-3' and reverse primer 5'-cgttgaaaacatataactcctgctc-3' |
| *H. sapiens TEN2* | Graumman et al, 2017 | Product size: 400/427 bp. Primer sequences: TGC TCT GTT GAA GTG TGC TCA and TGT TCT GAC AGG CTG ACT GC |
| *H. sapiens TEN2* | Graumman et al, 2017 | Product size: 560/350 bp. Primer sequences: AGA CTG GGA CTG CTG GTG ATT and ATC TGA CTC CGC CGA TTG G |
| *H. sapiens TEN4* | Graumman et al, 2017 | Product size: 250 bp. Primer sequences: CTG TGC TGC CGA CTG TGG TGG and AGT GCC AAC CAT TCA GGT CTA AG |
| *H. sapiens TCAP-2* | Torres-da-Silva et al, 2017 | 496bp: Forward primer 5'-gacaagatgcactacagcatcgac-3' and reverse primer 5'-ccatctcattctgtcttaaaaactgg-3' |

Supplementary material - Table 2: List of primers and probes used in the reviewed articles. The table presents the available information regarding primers, probes and related material used to identify teneurin DNA/RNA in several tissues cited in this review. The table presents the precise methodological data informed by the articles cited in the review.
